# Hardware‐Attentive Programmable Fourier Ptychography Enables Task‐Adaptive Label‐Free Virtual Staining

**DOI:** 10.1002/advs.76292

**Published:** 2026-06-30

**Authors:** Tianyue He, Wenyi Jing, Le Zhang, Qican Zhang, Tingdong Kou, Xin He, Yan Qiu, Yang Lu, Jian Cui, Yongfu Wen, Zhenrong Zheng, Hongying Zhang, Dayong Jin, Junfei Shen

**Affiliations:** ^1^ College of Electronics and Information Engineering Sichuan University Chengdu Sichuan China; ^2^ Department of Pathology West China Hospital Sichuan University Chengdu Sichuan China; ^3^ Institute For Biomedical Materials and Devices (IBMD) Faculty of Science University of Technology Sydney Ultimo New South Wales Australia; ^4^ Jiangxi Gaorui Optoelectronics Co., Ltd Nanchang Jiangxi China; ^5^ State Key Laboratory of Modern Optical Instrumentation College of Optical Science and Engineering Zhejiang University Hangzhou China

## Abstract

Virtual staining will enable fast and reagent‐free histological imaging toward accurate and consistent pathology diagnosis. However, most current developments rely on heuristic optical landmarks and blind reconstruction, which reduces interpretability, limits cross‐task adaptations, and prevents their ultimate clinical translations. Here, we introduce an interpretable Plain‐to‐Stain framework, “Task‐Adaptive Programmable Optics (TAPO)” that removes manual landmark selection by directly tying programmable optical control to task‐specific diagnostic objectives. The approach operates under specially modulated visible light, and the highly programmable imaging system decomposes each optical step to execute optics‐attention‐based reconstruction, leading to task‐adaptive multimodal optical encoding for virtual staining. We demonstrated that TAPO successfully passed rigorous validation across diverse tissue types (liver, lung), pathological states (normal, malignant), staining protocols (H&E, Masson's trichrome), magnifications (10×, 20×), and section‐thickness settings including routine 4 µm sections and moderately thicker 10 µm sections. Blinded assessments by a panel of board‐certified pathologists on multiple virtual slides showed that TAPO results had high clinical acceptance, especially in resolution, cytoplasmic clarity, and nuclear staining, and demonstrated considerable potential for clinical pathology practice.

## Graphical abstract

Task‐adaptive programmable optics enables label‐free virtual staining through optical‐attention‐guided acquisition and reconstruction. By optimizing wavelength, illumination angle, exposure time, and imaging depth, the framework learns task‐relevant optical measurements, generating clinically interpretable virtual stains with improved fidelity, non‐phototoxic visible‐light acquisition, and compatibility with standardized pathology workflows.

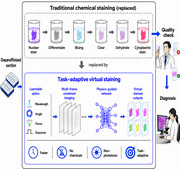

## 1 Introduction

Histopathological diagnosis provides essential information for disease management. Hematoxylin and eosin (H&E) staining is considered the gold standard for microscopic examination of tissues in histopathology [1]. The typical H&E staining procedure includes deparaffinization, rehydration, hematoxylin and eosin staining, dehydration, clearing, coverslipping, and microscopic imaging [2], which is labor‐intensive and time‐consuming, and can be affected by protocol differences, reagent batches, staining time, and operator‐dependent variations [3, 4]. The stained results can be easily affected by multiple factors, such as reagent concentration, staining time, solution temperature, pH value, and differences in staining procedures. It is widely recognized as a challenge to maintain consistent staining quality for accurate pathological diagnosis [5]. Non‐standardized staining results can undermine the diagnosis reliability or delay critical diagnostical results [6]. In the era of digital pathology, variability significantly hampers the need for rapid generation of standard digital pathology images, which are crucial for reproducible diagnosis across hospitals and large‐scale computational pathology [7, 8].

Recent advances in deep learning offer a promising solution by directly generating histological stains from unstained input, thereby reducing dependence on conventional chemical staining workflows and improving the efficiency and standardization of pathology workflows [9, 10, 11]. Known as virtual H&E (VHE) staining, the computationally derived stains from label‐free microscopic images [12, 13, 14] can be inherently standardized for enhancing diagnostic reproducibility [15, 16]. To date, **stain‐to‐stain methods** and **prestain‐to‐stain methods** are the two distinct technological categories. The former converts images of stained tissue samples (e.g., H&E or fluorescent‐stained) into other types of stains (e.g., Masson's trichrome and immunohistochemical staining, etc.) [10, 17, 18]. H&E‐stained tissue images have been successfully converted into a virtual periodic acid‐Schiff (PAS) stain [18], which can be converted into Acid Fuchsin Orange G (AFOG), CD31 IHC, and Collagen III (Col3) stains [19]. These transformations enabled the segmentation accuracy comparations across different stain types within the same field‐of‐view (FOV), which is difficult to achieve by traditional histology, as a single tissue section can be stained with only one type of stain [20, 21]. The latter, prestain‐to‐stain imaging, refers to optical or molecular imaging approaches that extract specific cellular structures before chemical staining, typically by exploiting intrinsic contrast mechanisms such as fluorescence, vibrational scattering, absorption, polarization‐related vectorial optical contrast, and mass‐spectrometry‐based molecular contrast [22, 23, 24, 25]. For example, polarization‐ or birefringence‐related imaging can provide additional anisotropic structural information for virtual histology [26, 27], while imaging mass spectrometry can provide highly multiplexed molecular distributions that help link molecular maps with tissue morphology [28]. These studies indicate that prestain‐to‐stain imaging is not limited to conventional optical contrast, but can also incorporate vectorial structural information and molecularly resolved information. Photoacoustic imaging [29, 30, 31] captures signals from areas with strong optical absorption, such as blood vessels or melanin, so that the network will be able to recover vascular structures or pigmented regions in the final virtual stain. Autofluorescence imaging [9, 32, 33] uses the intrinsic autofluorescence from molecules like collagen, NADH, and FAD to reveal tissue metabolism and extracellular matrix patterns, which can guide the virtual staining of nuclei, cytoplasm, and connective tissue. Raman spectroscopy imaging detects the molecular vibrations of nucleic acids, proteins, and lipids, helping the model distinguish between different cell parts such as the nucleus, cytoplasm, and cell membrane [34]. By providing such structural or molecular cues, the network will have a clearer starting point to learn how to generate realistic and interpretable virtual stains.

Recently reported pre‐stain methods usually rely on fixed optical contrasts or standard RGB bright‐field measurements, which can provide clear morphology for routine pathology but may not always expose sufficient task‐relevant information for label‐free virtual staining [35, 36]. The arbitrarily chosen optical markers that differ across staining tasks make cross‐task generalization difficult. Moreover, when virtual staining is learned only from conventional bright‐field intensity images, the network may rely predominantly on image‐to‐image appearance mapping and data‐driven correlations from training pairs, while physically informative cues for tissue‐component discrimination are still weakly expressed in the source measurements [37]. From an information‐theoretic perspective, this “arbitrary markers + blind reconstruction” stubbornly lacks interpretability, and because it cannot show which hardware settings or algorithmic components actually provide the diagnostic value [38, 39], clinicians will soon lose their confidence. As a result, standard intensity‐only acquisition may not always expose sufficiently informative cues for virtual staining, especially when subtle tissue and cellular constituents need to be distinguished [34]. When the source measurements contain limited task‐relevant information, reconstruction may depend more heavily on statistical appearance mapping [14, 40, 41], which can reduce fidelity and increase the risk of unreliable synthesis or hallucination [42, 43]. Moreover, high‐intensity laser excitations are often required, causing samples’ photodamage and photobleaching, and the weak emissions and spectral overlaps of biomolecules often challenge the stability and the high contrast of the inputs.

**FIGURE 1:**
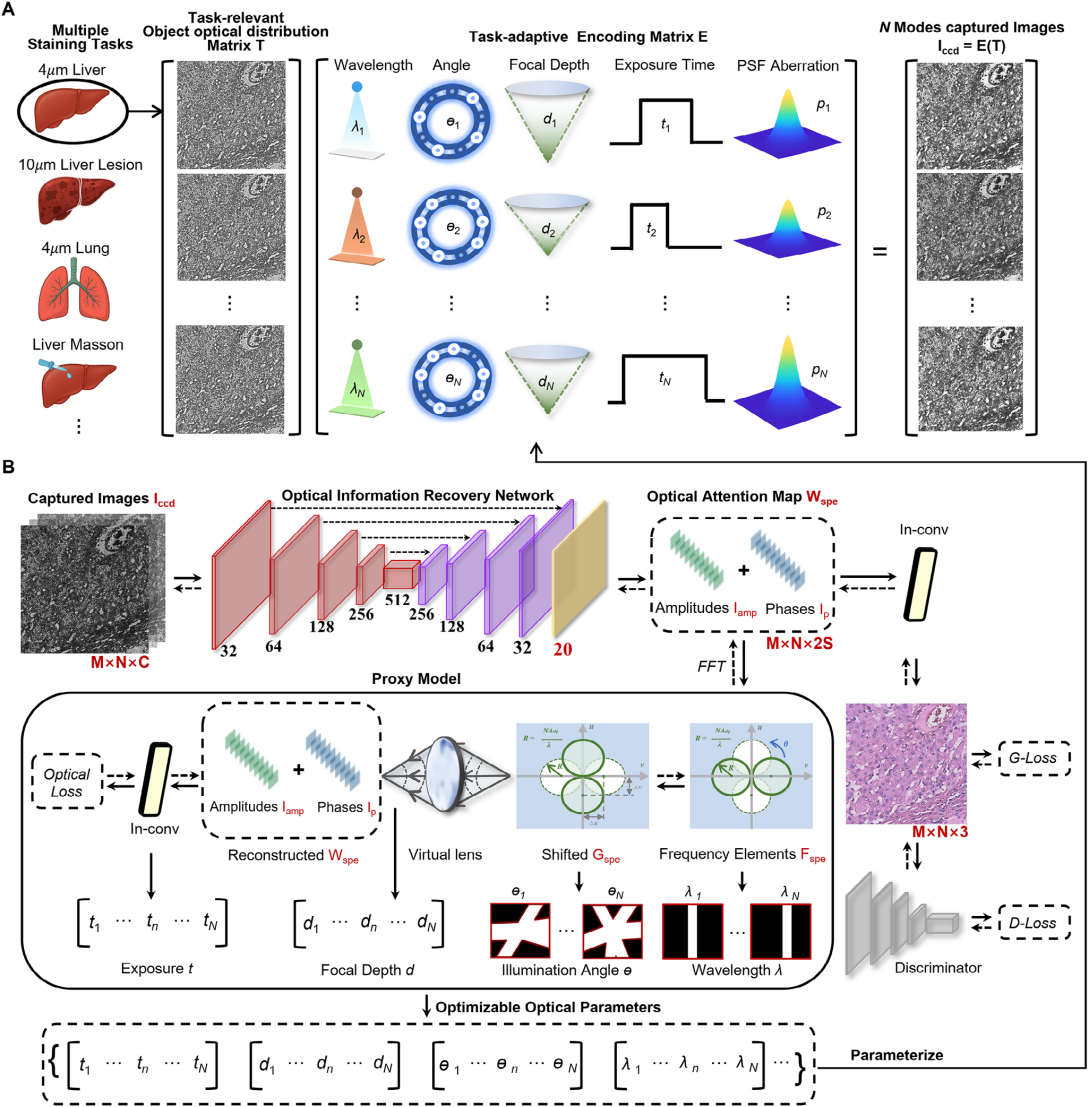
Task‐adaptive computational microscopy with learnable optical encoding and closed‐loop decoding. (A) Task‐adaptive optical encoding. T denotes the task‐relevant object matrix that represents tissue optical‐property distribution, and E denotes the optical encoding matrix that defines how the tissue object is encoded under different optical conditions. The interaction between the task‐relevant object matrix T and the optical encoding matrix E represents the optical imaging process under different encoding modes, where different encoding matrices encode the tissue object in different ways and generate the corresponding input images for the downstream virtual staining model. (B) Closed‐loop decoding and optimization. An optical information recovery network reconstructs the complex‐wave attention map from raw intensity inputs, and a differentiable optical proxy simulates each step of the real optical propagation process to reconstruct input images. The In‐conv layer transforms the reconstructed wavefield into a diagnostic virtual stained image, while the decoding results provide feedback to update the optical encoding parameters, forming a task‐adaptive hardware–software co‐optimization loop.

Here, we propose a plain‐to‐stain framework that performs task‐adaptiv optical encoding through hardware‐level optics‐attention optimization, with the goal of providing more informative label‐free measurements for downstream virtual staining reconstruction (as illustrated in Figure 1). We designed and built a microscopy system featuring a two‐stage spatial light modulation scheme, capable of programmable, high‐speed control over multimodal optical variables including spectral/angular illumination, exposure time, and imaging depth. This setup provides the physical foundation for implementing hardware‐level optics‐attention. Instead of exhaustively sampling the full spectral, angular, and phase‐related measurement space, the proposed framework treats programmable spectral selection, multi‐angle illumination, exposure control, and imaging‐depth adjustment as learnable optical variables, allowing the system to selectively acquire task‐relevant measurements and reduce the burden on downstream reconstruction. The learned optical parameters are task‐related rather than randomly determined during use, because each tissue/staining task is optimized using many fields of view from the corresponding tissue slides until the overall optical loss and staining loss converge within a small fluctuation range. To enable end‐to‐end differentiability for hardware attention, we developed a physics‐informed virtual lens proxy that simulates each optical step and co‐optimizes with the network, which realizes self‐calibration of imaging parameters and task‐adaptive optical encoding of the clinically relevant features. Unlike pre‐stain methods, this framework learns modulation policies directly from staining objects without requiring pre‐stain contrast design, rendering the method adaptive to various staining tasks. In our approach, the system is immune to photodamage, enabling targeted information extraction and optical encoding from a broadband coherent optical field, and the imaging chain is decomposed into explicit stages, producing interpretable “optical saliency” maps that can reveal which parameter drives the clinically relevant information toward precise diagnosis. In a practical workflow, the proposed virtual staining step is introduced after standard section preparation and operates directly on unstained slides, without changing upstream procedures such as fixation, embedding, sectioning, or slide preparation, as shown in Figure S9.

## 2 Results

### 2.1 Task‐Adaptive Computational Imaging System With Learnable Optical Controls

The proposed framework establishes a multi‐task adaptive imaging paradigm, where each pathological task is first represented as a task‐relevant object matrix **T**. This task matrix interacts with a set of learnable optical encoding matrices **E**, each element corresponding to one physical control dimension: wavelength, illumination angle, focal depth, and exposure time. These matrices form a structured encoding block [*t*_1_, …, *t_N_*], [*d*_1_, …, *d_N_*], [*θ*_1_, …, *θ_N_*], [*λ*_1_, …, *λ_N_*]. For this purpose, we designed a dual‐DMD computational illumination system (Figure S4) that provides independent and programmable control over four key parameters: wavelength (DMD1), angular direction (DMD2), imaging depth (annular illumination), and exposure time (CCD). Spectral modulation enhances sensitivity to biochemical composition, angular modulation captures boundary and fiber orientation, depth control compensates for tissue thickness variations, and adaptive exposure maintains an optical attention weight for each capture. Hardware implementation details are provided in section 3. This multi‐parameter encoding allows the microscope to adaptively project different optical patterns onto each capture, forming a task‐encoding response matrix **I** of captured intensity images.

Within this process, the encoded light fields are captured as intensity‐only measurements, forming a stack of captured images (**I_ccd_**) under multiple encoded conditions. These images are fed into the optical information recovery network, which reconstructs a complex‐valued wave representation (**W_spe_**), which serves as a latent bridge between low‐level optical inputs and high‐level semantic outputs. To maintain interpretability, an optical proxy module embedded within the network simulates the forward imaging process governed by the four hardware parameters. It maps the **W_spe_** into reconstructed input images **I_in_** and then introduces an optical loss that enforces physical consistency to real captured images **I_ccd_**, anchoring the learning to real optical behavior. During training, this module dynamically updates the original encoding matrices, refining the parameter sets [*t*_1_, …, *t_N_*], [*d*_1_, …, *d_N_*], [*θ*_1_, …, *θ_N_*], [*λ*_1_, …, *λ_N_*] through backpropagation. In this way, the decoding stage generates a new matrix representation, which feeds back into the encoding stage, forming a closed‐loop co‐optimization between the physical hardware and the computational model.

In parallel, **W_spe_** is mapped into a virtually stained image **I_out_** through an In‐conv module supervised by a GAN‐based loss. The generator minimizes perceptual and pixel‐level differences relative to chemically stained references, while the discriminator enforces visual realism and structural consistency. Compared with conventional CNNs, vision transformers, and diffusion models, this adversarial strategy offers sharper textures and more realistic staining patterns, while reducing risks of overfitting and hallucinations. Task‐adaptive framework implementation details, network architecture, and end‐to‐end training algorithm are given in section 3.

By jointly optimizing the physics‐informed proxy loss and the virtual staining loss, the proxy pathway constrains the learned wavefield to remain physically realizable and structurally faithful, while the staining branch drives it toward diagnostic discriminability. This mutual supervision enforces alignment between physical information and pathological semantics, allowing the system to dynamically balance interpretability, fidelity, and clinical relevance.

### 2.2 Performance Validation of Proxy‐Optimized VHE

**FIGURE 2:**
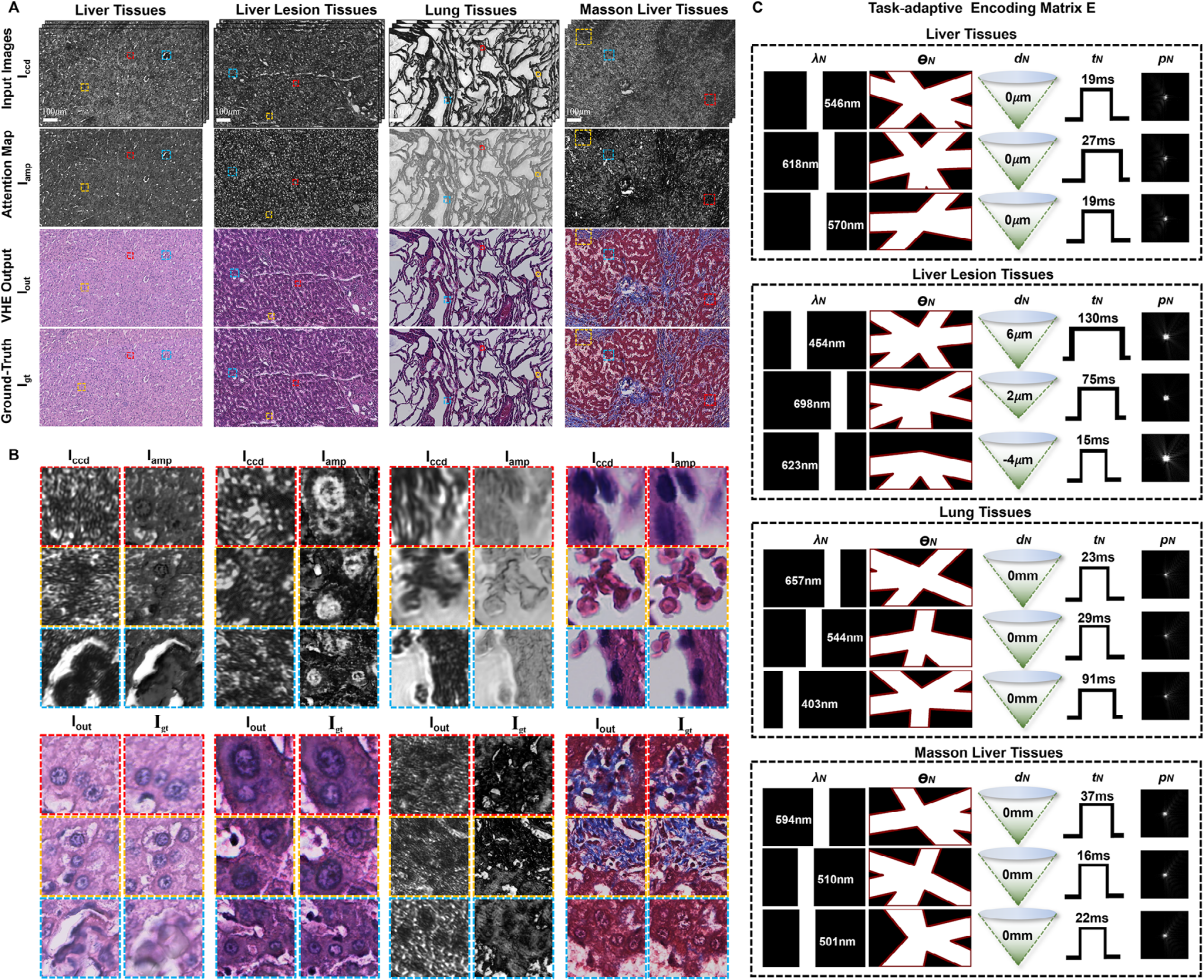
Experimental validation of task‐adaptive optical encoding across diverse histopathological conditions. (A) Representative results showing label‐free inputs, intermediate attention map, virtually stained outputs, and corresponding optimized optical parameters for different tissue conditions. Each column represents distinct imaging tasks, including variations in organ type, tissue thickness, stain type, and disease state. (B) Zoomed‐in details of the image results. C. Learned optical configurations from different tasks, demonstrating the system's ability to physically adapt its encoding strategy to each task for optimal reconstruction and virtual staining fidelity.

We evaluated TAPO on unseen liver tissue sections. Using 10×/0.1 NA images of 4 µm slices, TAPO transformed the raw unstained inputs into VHE with faithful nuclear and extracellular features (Figure 2A, first column). The outputs preserved polygonal hepatocytes, centrally located nuclei, sinusoidal spaces, and hepatic plate architecture, while enhancing high‐frequency details compared with chemical ground truth. Nuclear‐cytoplasmic contrast was sharpened through complex amplitude reconstructions, providing physically meaningful priors for virtual staining. The optical attention map **I_amp_** preserves the morphological structure of the input tissue while emphasizing the spatial positions of nuclei with high precision. This selective highlighting is crucial for guiding the subsequent virtual staining process and provides enhanced interpretability for the decoding stage by linking physical features to histological semantics.

Unlike conventional approaches relying solely on stained supervision, TAPO, based on a dual‐constraint strategy, integrates unstained raw inputs and a physics‐informed proxy. This yields VHE images that are both physically consistent and clinically interpretable (see detailed discussions of the intermediate results for all the experiments in Supplementary 1). Quantitative evaluations confirmed alignment with histopathology, for instance, the nuclear statistics, including the count, diameter, and spacing, clustered with ground truth, RGB violin plots showed near‐perfect overlap in hematoxylin‐sensitive blue channels, and metrics achieved KID = 0.0071 [44], FID = 48.08, and PSNR = 17.73 (Figure S1). Optimized proxy parameters (Figure 2B) selected informative wavelengths at 546, 570, and 618 nm, directional illumination masks, and co‐adapted exposure/imaging distances, producing virtual lens profiles with defocus/astigmatism correction and sharper PSFs.

To reveal fine histological details, such as chromatin texture and nucleoli that are critical for nuclear grading and subcellular abnormality detection, we further challenge 20× magnification that offers ∼4× higher spatial sampling. Though the smaller field of view reduces photon budget and increases frame count, leading to lower signal intensity and greater noise sensitivity, that further requires longer acquisition time. At 20× magnification, TAPO revealed fine nuclear textures and nucleoli critical for grading (Figure S2).

### 2.3 TAPO for 10µm Thickness Lesion Tissue

We next evaluated TAPO on 10 µm liver lesion sections, where increased tissue thickness may preserve more pathological information but can also lead to less uniform chemical staining. (Figure 2A, second column). Routine 3–4 µm slices reduce optical ambiguity but may truncate cells or nuclei during section preparation, whereas 10 µm sections have a higher chance of preserving more complete cellular and nuclear structures [45], as shown in Figure S10. To address this optical challenge, we introduced imaging distance (Δd) as a learnable parameter to enable dynamic focal‐plane adaptation and correction of depth‐induced aberrations. To achieve this, we employ a deep‐focus pyramid fusion algorithm to integrate the 5 focal layers and generate a large depth‐of‐field ground‐truth image (see section 3). The reconstructed VHE captured pathological hallmarks that are difficult to visualize in thin sections, including anisotropic chromatin distribution and crowded nuclear architecture. For chemically stained 10 µm sections, dye penetration, staining/dehydration time, and local processing conditions may vary across tissue depth and local regions, which can cause uneven staining appearance [46]. The corresponding virtual staining result shows a more uniform stain‐like appearance across the tissue field, supported by spectral and phase‐related cues and by learning from a large number of paired samples. Quantitative analyses (Figure S3) confirmed that nuclear‐level metrics, including counts, diameters, and spacing, clearly separated malignant from normal regions, with VHE outputs closely matching the ground truth. Cancerous nuclei appeared more numerous, larger, pleomorphic, and densely packed, consistent with diagnostic criteria.

Optimized proxy parameters (Figure 2B) revealed adaptive adjustments for thick tissue. For example, nonzero Δd values simulated defocus to reconstruct multiple in‐focus layers; exposure times tuned per wavelength enhanced SNR in deeper regions; and learned PSFs exhibited broadened lobes favoring multilayer integration. These adaptations preserved structural continuity and improved contrast of malignancy‐associated features at different depths. In Figure S3, RGB violin plots further showed strong overlap with the chemical staining results, supported by low KID (0.0025), FID (48.63), and PSNR (18.57 dB). Together, these results demonstrate that TAPO supports high‐quality virtual staining of thick sections, enabling accurate tumour boundary delineation, infiltration depth assessment, and staging in clinically challenging scenarios.

### 2.4 TAPO for Multiple Tissue Types (Lung Tissue)

Lung tissues, featuring with fragile and porous microstructures with thin alveolar walls, sparse capillaries, and delicate interstitial fibers (Figure 2A, third column), are prone to attenuation during staining and distortion under incoherent illumination, often causing boundary ambiguity. Clinically, the ability in resolving the fine features is crucial for tasks such as early interstitial lung disease detection, microvascular involvement assessment, and evaluation of the alveolar‐capillary interface in infection or fibrosis. TAPO preserved the fine features with high fidelity, such as delineated alveolar septa, resolved bronchiolar lining cells, and sharpened peripheral vascular contours. Moreover, the insets revealed the enhanced membrane contrast and chromatin texture, demonstrating biologically meaningful recovery of the low‐contrast coherent features. We reason that both contextual cues and physics‐informed proxy are the two complementary mechanisms that underpin such robustness achieved by TAPO, as the physics‐informed proxy enforces consistency with the unstained input.

The optimized optical parameters for lung tissue include three distinct wavelengths (657, 544, 403 nm), and the directional frequency supported by the optimized DMD2 patterns and the moderate exposure times (23, 29, and 91 ms) to avoid saturation of weakly stained boundaries. Compact and centered PSFs indicate a preference for minimal spatial blur, which improves the feature's edge sharpness. The achieved PSNR of 22.75 and 0.0048 KID, consistent with previous H&E results, demonstrates robust preservation of pulmonary microstructures. The intrinsic physical differences among tissue structures enhance the contrast beyond intensity‐only imaging. TAPO's learnable proxy constraints ensure tissue‐specific encoding to maintain structure consistency with the underlying optical measurements.

### 2.5 Extension to Masson's Trichrome Staining

TAPO can be extended for virtual Masson's trichrome staining, which shows connective tissue and fibrosis with high chromatic contrast (Figure 2A, last column). Masson staining emphasizes extracellular matrix components to enhance the diagnostic value, particularly in assessing liver diseases such as cirrhosis and steatohepatitis. More importantly, multiple complementary stains generated from the same tissue section ensure perfect spatial alignment, avoiding tissue loss during the physical multiple re‐staining process. The digitally generated Masson staining of liver tissue successfully detected collagen deposition in fibrotic areas, such as perisinusoidal and portal tracts, while maintaining clear boundaries between fibrotic and non‐fibrotic regions. Subcellular details, including nuclear morphology, sinusoidal capillaries, and Kupffer cells, were all well preserved.

Here, TAPO favors blue‐green spectral bands (594, 510, 501 nm), learns angular encodings that support fine frequency decomposition, with exposure times (37, 16, 22 ms) are adjusted selectively to balance signal across absorption‐dominant channels. The PSFs in this task are the narrowest among all (using 80% PSF energy distribution radius as comparison criterion), enabling fine‐grained reconstruction of color transitions and enhancing staining uniformity across tightly packed tissue structures. In the more chromatically complex Masson task, the network maintains low perceptual divergence (FID 57.14, KID 0.0037) and shows reliable generalization to alternative staining protocols.

### 2.6 Comparative Study

**FIGURE 3:**
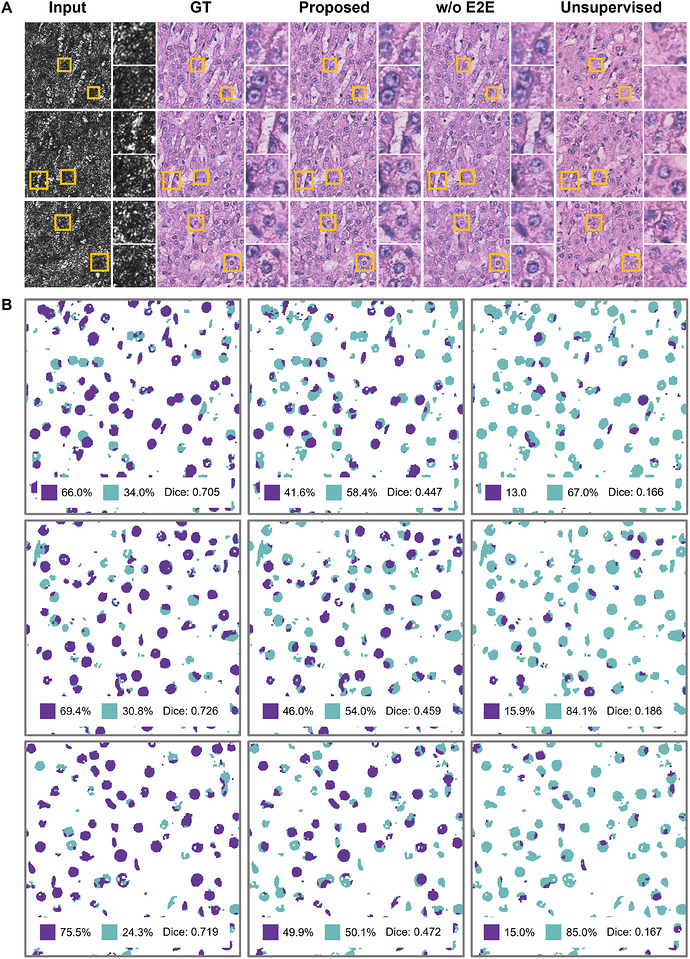
Comparative evaluation and pathologist blind evaluation of virtual staining methods. (A) Comparison experiments were conducted using three different strategies: our proposed end‐to‐end soft–hardware co‐optimized method, a non‐end‐to‐end baseline with fixed illumination patterns (w/o E2E), and an unsupervised learning approach trained on unaligned data. The proposed method produces clearer nuclear morphology, more consistent chromaticity, and finer cytoplasmic structure. (B) Cell nucleus localization analysis using automated segmentation. Predicted nuclei correctly overlapping with the ground truth are shown in purple; missed or mislocalized predictions are shown in green. The results reflect each method's capacity to preserve spatial coherence and high‐frequency morphological features.

We benchmarked our framework against two internal baselines, including a non‐end‐to‐end model with fixed random illumination and an unsupervised CycleGAN‐based approach (Figure 3). All experiments used 4 µm liver tissue sections captured with a 20× objective. As shown in Figure 3A, our method produced more consistent color tones, sharper nuclear morphology, and better subcellular preservation. In contrast, the w/o E2E baseline underperformed in fine detail recovery, while the unsupervised model failed to capture accurate structural boundaries due to domain mismatch. Automated nuclear localization (Figure 3B) further quantified reconstruction quality. Our method achieved the highest Dice score (0.713), compared to 0.467 for w/o E2E and 0.169 for the unsupervised model. These results demonstrate our framework's ability to retain high‐frequency, spatially coherent features essential for segmentation, enabled by task‐aware adaptive illumination.

Unlike baselines, our supervised end‐to‐end design propagates gradients through both optical parameters and network weights, enhancing spectral contrast and detail fidelity. This allows precise learning of nuclear size, shape, and spatial distribution, resulting in superior segmentation performance. The non‐end‐to‐end baseline exhibits moderate structural stability but lacks adaptive control over illumination parameters, resulting in limited contrast and weakened reconstruction of fine details. The unsupervised approach retains overall color style but fails to capture precise morphological features and localizations due to the lack of spatial alignment during training. Collectively, these findings confirm that end‐to‐end, physics‐informed learning is critical for producing virtual stains that are both visually faithful and diagnostically informative.

In, Table S1, we further added a broader literature comparison with representative published virtual staining methods based on unstained bright‐field microscopy [47, 48, 49], label‐free photoacoustic histology [30], autofluorescence microscopy [26], FLIM [50], and other enriched‐input settings. The summarized results show that the proposed framework achieves the best SSIM and the lowest reported FID in this comparison, indicating strong structural fidelity and distribution‐level realism.

### 2.7 Pathologists' Overall Clinical Evaluation

**FIGURE 4:**
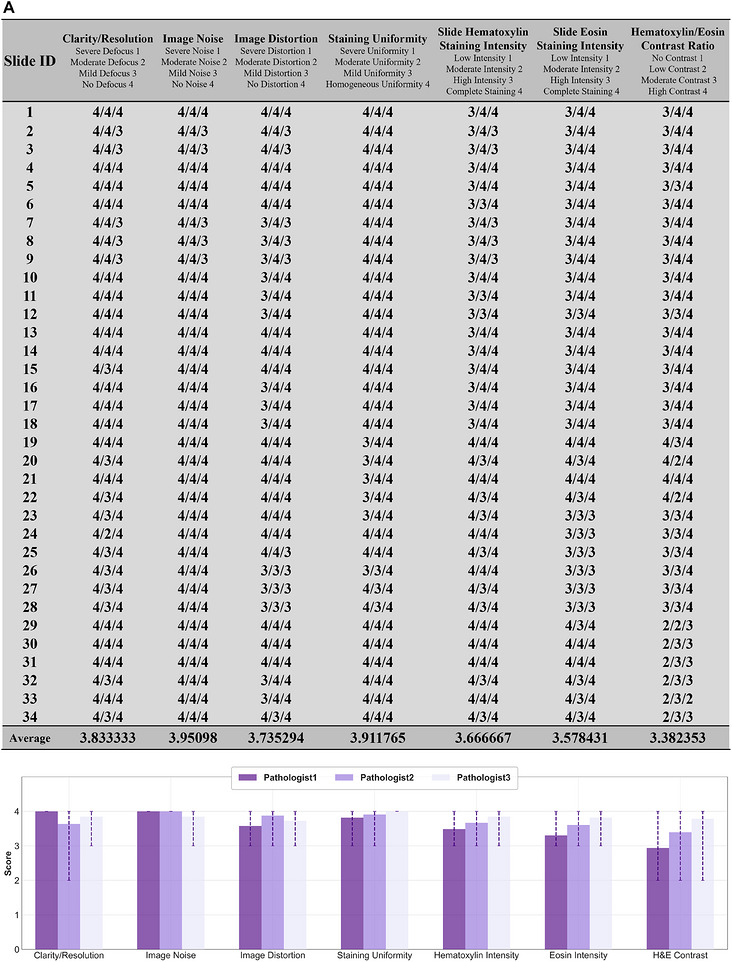
Pathologist‐based diagnostic evaluation highlights structural fidelity and clinical reliability of the virtually stained results. (Top) A blinded assessment was conducted by three board‐certified pathologists across 34 virtually stained whole‐slide tissue images. Each case was rated on a four‐point scale (1 = poor, 4 = excellent) by pathologists 1/2/3 based on seven histopathological criteria: clarity and resolution, image noise, geometric distortion, staining uniformity, hematoxylin intensity, eosin intensity, and nuclear–cytoplasmic contrast. These dimensions reflect core diagnostic needs such as nuclear detail, cytoplasmic delineation, and chromatin‐based differentiation. (Bottom) The bar chart summarizes the averaged diagnostic ratings across three pathologists.

To validate diagnostic applicability, we designed a set of evaluation criteria as described in Supplementary 1, and conducted a blind evaluation with three board‐certified pathologists on 34 virtually stained whole‐field images (Figure 4A). Each image was scored from 1 (poor) to 4 (perfect) across clarity, noise, distortion, staining uniformity, hematoxylin intensity, eosin intensity, and contrast. TAPO achieved consistently high ratings in terms of clarity (3.83) that indicates sharp subcellular details with minimal defocus, noise (3.95) and distortion (3.73), that reflects preserved structural integrity with only minor artifacts, and staining uniformity (3.91) that confirms stable color distribution across fields. Hematoxylin (3.67) and eosin (3.58) intensities showed slight variation, suggesting effective nuclear‐cytoplasmic differentiation with room for contrast optimization. The H&E contrast ratio (3.38), although the lowest, demonstrated balanced chromatic separation between nuclei and cytoplasm. Despite minor inter‐rater differences, trends were consistent among the three pathologists, particularly in uniformity, nuclear contrast, and overall clarity, which confirms that virtually stained images meet the core visual and structural requirements for routine diagnostic interpretation.

### 2.8 Pathologists' Detailed Clinical Evaluation

**FIGURE 5:**
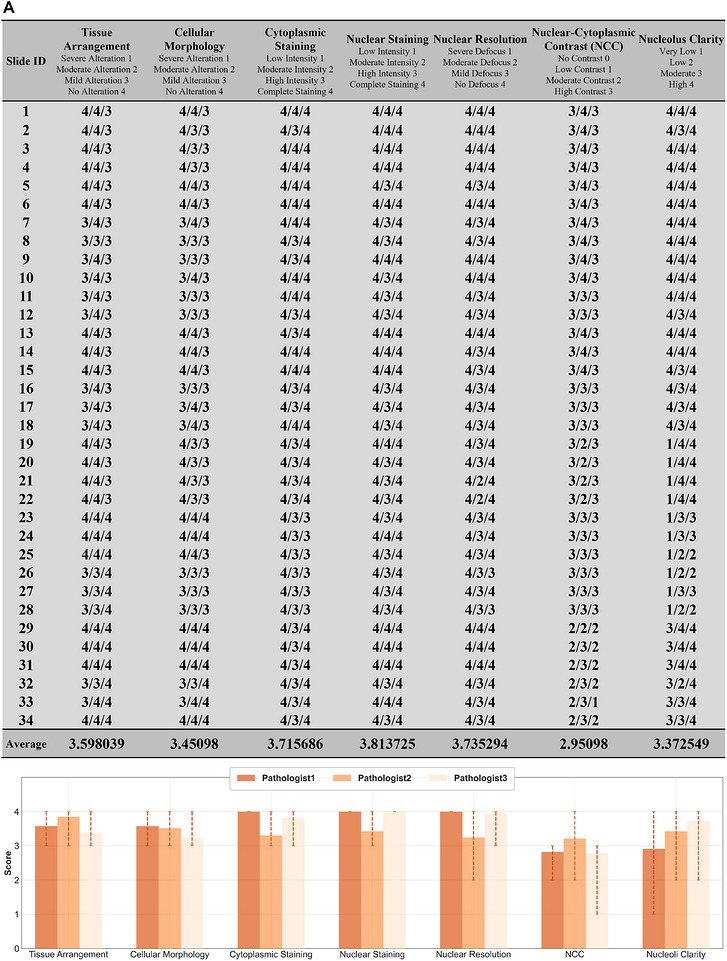
Detailed pathologist evaluation confirms preservation of fine‐grained morphological features essential for diagnostic interpretation. (Top) Three board‐certified pathologists independently assessed 34 virtually stained tissue sections based on seven structure‐specific criteria: nuclear staining, cytoplasmic staining, nuclear resolution, tissue arrangement, cellular morphology, nuclear–cytoplasmic contrast (NCC), and nucleolus clarity. (Bottom) The bar chart shows consistent agreement among raters, with low inter‐observer variability across most categories.

An additional blind evaluation of clinical detail quality was conducted with three pathologists, who independently scored nuclear and cytoplasmic features, tissue arrangement, and fine structural details (Figure 5A). The highest scores were observed for nuclear staining (3.81), nuclear resolution (3.73), and cytoplasmic staining (3.72), confirming well‐preserved morphology and reliable contrast. Tissue arrangement (3.59) and cellular morphology (3.45) also showed strong consistency, indicating preserved spatial integrity across reconstructions. Fine‐detail metrics showed more variability. For example, nuclear‐cytoplasmic contrast (2.95) and nucleolus clarity were rated lower, particularly in lung tissue, reflecting intrinsic histological challenges such as low nuclear density and alveolar‐rich structures. Despite these subtleties, inter‐rater agreement was high, with most scores clustered at 4/4/4 or 4/3/4. These findings demonstrate that TAPO consistently preserves diagnostically critical features such as nuclear atypia, chromatin texture, and cytoplasmic contours, while maintaining global tissue architecture and supporting its integration into routine diagnostic workflows.

## 3 Methods

In this section, we detail the design and implementation of the proposed task‐adaptive optical encoding and decoding pipeline. This pipeline integrates a reconfigurable hardware illumination system with a neural algorithmic decoder to enable adaptive light‐field acquisition and high‐fidelity virtual staining. We first introduce the architecture of our computational illumination hardware, then describe the design of the deep neural decoding model, which reconstructs semantically rich, high‐resolution stained images from captured multi‐condition intensity inputs.

To bridge real‐world optics and differentiable learning, we incorporate a physics‐informed proxy model based on a virtual lens formulation. Details of the proposed proxy model are also given here. This module simulates the real forward imaging process with wavelength‐ and angle‐specific point spread functions (PSFs), enabling gradient‐based learning of physical parameters. Finally, we present the end‐to‐end soft‐hardware co‐optimization strategy, where optical parameters and network weights are jointly updated within a closed‐loop learning framework.

### 3.1 Implementation Details of Proposed Reconfigurable Optical Encoder

The detailed optical structure of the multi‐wavelength and multi‐angle annular imaging system is illustrated in Figure S4, which supports programmable control over wavelength, angular direction, exposure time, and imaging distance, allowing real‐time adaptation of optical coding to task‐specific demands. A white LED point light source is filtered through an aperture **A** to remove stray light, followed by a beam expansion and collimation lens group (**L1 and L2**) to achieve uniform, wide‐field illumination. The resulting uniform light is spectrally dispersed via a diffraction grating **G** and enterers a Fourier lens **L3**; as the grating is positioned at the focal plane of the Fourier lens, the output light with different wavelengths is parallelly projected into distinct locations on the first spatial light modulator DMD 1 (Digital Micromirror Device). The illumination pattern of DMD 1 is shown in Figure S4, including a rectangular continuous spectrum across the full visible spectral range. The DMD 1 enables spectral selection by allowing one or several wavelengths to pass, effectively controlling both spectral band selection and bandwidth adjustment. For demonstration, the red and purple lights are allowed to pass. Only pixels with a value of 1 (dark gray pattern) allow light to pass through, while those with a value of 0 (light gray pattern) filter light out. After selecting specific spectral bands, the output light passes through a 4f system containing a conical lens group, which consists of two convex lenses (**L4** and **L5**) and two cone lenses (**C1** and **C2**). By leveraging the air gap between the cone lenses, this system forms an annular illumination output to a specific radii size *R*. By adjusting the air gap between the lenses, different radii of annular illumination can be achieved, thus controlling the range of transmitted illumination. After a relay lens **L6**, the annular illumination light subsequently reaches the second spatial light modulator DMD 2, which dynamically adjusts the angle of incidence to achieve sequential alternation across 360° angles, enabling uniform multi‐angle illumination. The DMD 2 is placed at the front focal plane of a convex lens **L7**, realizing far‐field illumination. The final selected beams pass through the sample at the focal plane of the objective lens (**OBJ**). A motorized rotary stage is used to enable imaging at different magnifications with various objectives. An **XYZ stage** is employed for scanning of the sample. After passing through a tube lens (**T**), the imaging result is captured by a CCD and transmitted to the computing center.

### 3.2 Optical System Hardware Specifications

Our system employs a high‐intensity coaxial single white LED illuminator (Advanced Illumination), compatible with 8 mm or 1.4“ fiber interfaces, providing coverage of the visible spectral band. The beam conditioning module (L1 and L2) consists of plano‐convex lenses (5 and 50 mm diameters, EFL = 5 and 50 mm respectively, N‐LASF14/N‐BK7 substrates) for collimation and expansion. A 300 grooves/mm diffraction grating (50 mm square, 17.5° blaze angle) disperses the light, followed by a 100 mm diameter plano‐convex lens L3 (EFL = 200 mm, N‐BK7) for primary focusing. The system incorporates two DLP65DCFYE DMD (0.65”, 1920 × 1080) for spatial modulation, with relay optics using matched 50 mm plano‐convex lenses (EFL = 50 mm) in a 4f configuration. The imaging path terminates with a finite conjugate objective with configurable magnifications and MT‐1 tube lens (EFL = 200 mm). All refractive elements feature uncoated surfaces with 40‐20 surface quality.

For computational processing, the system is supported by an AMD EPYC 9754 128‐Core Processor (Zen 4 architecture, 128 cores/256 threads) and an NVIDIA GeForce RTX 4090 GPU (24GB GDDR6X, 16,384 CUDA cores), ensuring high‐performance data handling and real‐time image processing capabilities.

### 3.3 Image Dataset Preparation

The dataset used in this study contains 105 patients, 115 corresponding slides, and over 35,000 groups of images for normal liver, lesion liver, lung, and Masson‐stained liver tasks. Each task uses the full‐slide image from its corresponding specimen, and the train/validation/test split is set to 8:1:1 at the slide level. In the current implementation, each sample is acquired with 3 raw frames, and each raw frame is obtained under a specific optimized optical encoding condition that includes spectrally selected continuous 3 broadband and 6 multi‐angle illuminations encoding. The raw image captured by a single camera shot has a size of 4800 × 2880 pixels and is cropped into 960 × 960 patches for network training; with a camera pixel size of 2.4 µm, the corresponding physical field of view is 1.15 × 0.69 mm at 10× magnification and 0.58 × 0.35 mm at 20× magnification. During training, one tissue slide is divided into multiple fields of view, and the model learns the virtual staining process from these fields of view while jointly updating the optical encoding parameters and the reconstruction network. The summary of dataset composition and image acquisition settings is given in Table S2. The workflow for paired‐data generation and full‐field scanning, as well as the chemical staining workflow used for obtaining the stained ground‐truth data, are given in Figures S11 and S12, respectively.

### 3.4 Task‐adaptive Virtual Staining Framework Implementation Details

We design an adversarial decoding pipeline guided by a physics‐informed optical proxy model, enabling task‐adaptive reconstruction of coherent optical fields and virtual staining. The generator optical information recovery network receives multi‐frame encoded inputs and reconstructs a coherent wavefield representing the sample's coherent transmission function. This spectral distribution, or complex amplitude, encapsulates both the amplitude and phase characteristics of the tissue, providing structural and refractive information that is not directly accessible from conventional bright‐field intensity measurements alone and is therefore useful for distinguishing subtle tissue constituents during virtual staining [24, 51, 52]. Recovering such a complex field can be viewed as a typical inverse problem. The use of multiple input frames reflects distinct optical modulations applied to the same underlying sample, each introducing a different constraint to the inverse problem. In essence, each modulation acts as a unique equation in a system that solves for the unknown complex transmittance. Our task‐adaptive framework adaptively selects illumination conditions that are analogous to linearly independent measurements, thereby maximizing encoding diversity and improving the conditioning of the inverse problem.

As shown in Figure 1B, the framework begins with a set of captured raw images obtained under varying optical conditions. The use of a single white LED source ensures partial spatial coherence in illumination, providing the necessary physical foundation for reconstructing complex‐valued coherent fields. Therefore, the optical information recovery network receives multiple intensity‐only inputs **I_ccd_** with the size of (**M**×**N**×**C**) and reconstructs a complex‐valued wavefield representation **W_spe_** (**M**×**N**×**2S**), where each spectral band **S** is encoded by two channels: amplitude Iamp and phase **I_p_**. This intermediate complex‐field representation **W_spe_** captures resource structural and spectral information underlying the tissue sample. The reconstructed complex‐valued wavefield should therefore be understood as a multimodal information carrier that bridges task‐adaptive optical encoding and virtual staining reconstruction.

### 3.5 Optical Imaging Modeling by Virtual‐Lens–Based Proxy Model

To physically constrain the reconstruction of high‐fidelity coherent wavefields, we design a virtual‐lens–based proxy model that mirrors the behavior of our real optical system. This proxy serves as a differentiable simulation of optical image formation, enabling loss backpropagation from the reconstructed input to hardware parameters. Compared with proxy models tied to a fixed optical structure or predefined PSF, the virtual‐lens‐based physics proxy provides a more flexible differentiable representation to fit system aberrations and unknown optical deviations while maintaining physical consistency. We begin by transforming the spectrally encoded complex field **W_spe_** (output of the optical information recovery network, Figure 1B) into the frequency domain via a Fourier transform. The obtained frequency component **F_spe_** is then multiplexed with a learnable rotation matrix ***R*** in the frequency domain to emulate the angular modulation introduced by DMD2. The core operation involves a trainable rotation matrix parameterized by an angle **𝛼**, which is initialized at zero and updated during training. The 2D rotation transformation is defined as ***R*[·]**, and the tensor after the rotation transformation is defined as **G_spe_**. Given input pixel (*x*, *y*) of **F_spe_**, the rotated pixel (*p*, *q*) of **G_spe_** can be calculated as:

(1) $$\left(\begin{array}{c} p\\ q \end{array}\right)=R\left[\left(\begin{array}{c} x\\ y \end{array}\right)\right]=\left(\begin{array}{cc} \cos\alpha & -\sin\alpha \\ \sin\alpha & \cos\alpha \end{array}\right)\left(\begin{array}{c} x\\ y \end{array}\right)$$

### 3.6 Virtual Lens PSF Formation Model

The **G_spe_** is subsequently filtered via linear convolution with wavelength‐specific PSFs generated by the virtual lens. For accurate PSF modeling, we specifically design a virtual lens (VL) model as a digital counterpart of the real physical optics. Unlike static lens imaging approximations, our VL model is designed to produce learnable wavelength‐ and depth‐specific PSFs, making it adaptable to a wide range of optical behaviors encountered in real imaging. For each illumination wavelength *λ*, we simulate wavefront propagation of **G_spe_** through a wavelength‐specific VL PSF, corresponding to DMD1's control of spectral selection. Also, the PSF is determined by the imaging distance ***d***, corresponding to the moving distance of the motorized XYZ stage. This hardware one‐to‐one mapping modeling leads to greater interpretability through explicit alignment with real optical parameters. The resulting PSF acts as a core component of our proxy model, enabling posterior‐constrained supervision by ensuring that the reconstructed wavefield produces realistic degraded measurements. Based on the optical diffraction model, the VL PSF is defined as:

(2) $$\begin{array}{ccc} & & PSF\left(x,y,\lambda \right)\\ & & \propto {\left|\frac{e^{ikd}}{i\lambda d}\int \int U_{p}\left(x_{p},y_{p},\lambda \right)e^{\frac{ik}{2d}\left[{\left(x-x_{p}\right)}^{2}+{\left(y-y_{p}\right)}^{2}\right]}dx_{p}dy_{p}\right|}^{2} \end{array}$$

where *k* = 2π/*λ* is the wave number, *λ* is the wavelength, *d* is the image distance of VL, (*x_p_*, *y_p_*) and (*x*, *y*) denote the spatial coordinates at VL and the real sensor plane. *U_p_* (*x_p_*, *y_p_*, *λ*) = *U*_0_(*x_p_*, *y_p_*, *λ*)*A*(*x_p_*, *y_p_*)exp[*iφ*(*xp, yp, λ*)] and *U*_0_(*x_p_*, *y_p_*, *z*, *λ*) = exp[*ik* (*x_p_* ^2^+*y_p_* ^2^+*z* ^2^)^1/2^] are the output wave filed and input aspheric wave of the VL, with *z* as object distance. *φ*(*x_p_*, *y_p_*, *λ*) = *k* ×*h*(*x_p_*, *y_p_*, *λ*) is the phase map introduced by the VL, with *h*(*x_p_*, *y_p_*, *λ*) representing VL height map, and *A*(*x_p_*, *y_p_*) is a **circ** function with discretized values of zero and one that represents the aperture of VL. The VL height map adopts a dual‐profile design: a fixed ideal thin‐lens height map base for guaranteed imaging formation, and a trainable Zernike polynomial expansion to simulate high‐order aberrations, defocus, and thermally induced disturbances. The ideal thin‐lens base ensures optimization stability during training as it follows a point‐to‐point imaging principle via a perfect PSF. Meanwhile, the posterior constrained supervision by recovered image Iin allows the system to implicitly learn unknown or difficult‐to‐model physical deviations, including imperfect alignment, thermal fluctuations, or lens manufacturing errors. Detailed derivation of Equation (2) is given in Supplementary 1. After spectral rotation and PSF modulation, we reconstruct the degraded input image by inverse Fourier transforming the convolved result. The recovered input image **I_in_** can be calculated as:

(3) $$I_{in}=F^{-1}\left\{R\left[G_{spe}\right]\times F\left(PSF\right)\right\}$$

where 𝓕 is the Fourier transform operator. To complete the simulation of physical exposure conditions, exposure time is also modeled in the proxy to reflect camera integration behavior. Together, these components form a physically grounded proxy of the full acquisition process, which replicates the real imaging process, reduces optical‐encoding uncertainty, and makes it controllable. The output of the virtual lens is processed by an In‐conv sub‐module and generates final proxy model output **I_in_**, where the In‐conv submodule applies a learned channel‐wise attention mechanism to reweight spectral‐phase channels and compress the tensor into an RGB format. Then, **I_in_** is compared against the original captured image **I_ccd_** to compute the optical reconstruction loss. This posterior loss is backpropagated through the neural network and proxy model, enabling gradient‐based updates to the four physical parameters and regularizing the latent representation to be consistent with the real physical image formation process. The detailed inner structure of the generator and discriminator is given in Supplement 1.

### 3.7 End‐to‐End Optimization Pipeline with Hardware Attention

Building upon the task‐guided optical encoding system and the proxy‐model‐based decoding framework, we further integrate both optical and algorithmic parameters into a unified end‐to‐end optimization loop to realize adaptive optical information encoding. This pipeline forms a hardware‐in‐the‐loop learning mechanism that enables task‐aware light field control during training. The process is summarized in Algorithm 1 (see Figure S5), which integrates both forward and backward propagation through the full imaging–computation loop. In the 10 um thickness experiment, we employ the deep‐focus pyramid fusion algorithm to integrate different focal layers (5 layers are used in our experiment) and generate a large depth‐of‐field ground‐truth image. The input of pyramid fusion (layer 1∼5) and corresponding output, as well as our reconstructed large depth‐of‐field results, are given in Figure S6.

At each training epoch, the system begins with a randomly initialized set of four learnable optical parameters: spectral band ***λ*** (controlled by DMD1), illumination angle ***ω*** (controlled by DMD2), camera exposure time ***t***, and imaging distance ***d*** (related to the motorized stage position). These parameters jointly determine the illumination configuration and sensing geometry for each image capture. Structured illumination patterns **I_dmd_** corresponding to the current ***λ*** and ***ω*** are loaded in two DMDs, and unstained raw images **I_ccd_** are acquired with the camera using the current exposure time ***t*** and sensor distance ***d***. These raw images are then registered to the corresponding chemically stained ground truth images to obtain aligned training pairs. The parameterized model takes these pairs as input and performs two decoding tasks simultaneously: it generates a virtually stained image **I_out_** and reconstructs the input **I_in_** by passing the recovered wavefield through a proxy imaging model. The training loss ***L*** is composed of two parts: (1) a generative loss comparing **I_out_** with the ground truth, and (2) a physical consistency loss measuring the similarity between **I_in_** and the actual measured input **I_ccd_**. The total loss is then backpropagated through both the neural network and the proxy model, allowing simultaneous updates to the model parameters ***θ*** and the four optical variables {***λ, ω, t, d***}. This pipeline embodies the concept of hardware attention, where the optical system is no longer a static data collector but an adaptive encoder that learns to emphasize diagnostically relevant features during acquisition. Unlike algorithmic attention, which operates on features after imaging, hardware attention is realized physically by dynamically learning different types of optical parameters. This enables the system to extract task‐guided information directly at the source, improving feature quality and relevance under limited optical bandwidth. By enriching the source measurements with spectral and phase‐related cues before reconstruction, the framework reduces the extent to which virtual staining must rely only on blind appearance mapping from standard intensity images. In scenarios with low signal levels or subtle morphological cues, this adaptive optical encoding enhances the efficiency of information capture, reduces the burden on downstream networks, and ensures that the acquired data is inherently aligned with the staining objective.

Although the acquisition strategy changes according to different tasks, the variation of learned optical parameters is controlled by the convergence criterion during training, where the optical encoding parameters are optimized together with the reconstruction network using many fields of view from the same tissue/staining task. In our experiments, convergence is determined when the change of overall optical loss and staining loss between two consecutive training rounds is smaller than 0.005, as shown in Figure S13. After convergence, repeated optimization under the same tissue type and staining task tends to produce similar optical configurations, indicating that the learned optical parameters are reproducible for a given task. This reproducibility is further supported by the physics‐informed proxy model, which constrains the optimized optical parameters and the reconstructed staining result to remain consistent with the physical imaging process and the input tissue structure. The robustness of the framework is supported by validation across data collected from different experimental batches and tissue preparation times, different tissue types (liver and lung), pathological states (normal and lesion tissues), staining protocols (H&E and Masson's trichrome), magnifications (10× and 20×), and section thicknesses (4 and 10 µm). Across these settings, the proposed method maintained stable virtual staining quality in tissue morphology, stain‐like color appearance, and structural fidelity, and the generated results were further supported by pathologist evaluation, indicating that the learned encoding strategy is strongly robust to varied acquisition conditions and application scenes. For different tissue types or staining objectives, the learned optical parameters can be inherited as initialization and then locally fine‐tuned to reach the optimal performance for the new tissue/stain setting.

In the current system, the acquisition time depends on the task and on the optimized exposure settings; for example, the average acquisition time is about 33 ms per frame in the 10× liver tissue experiment and about 43 ms per frame in the 20× liver tissue experiment. The training time is about 9 h for one task, and the inference time is about 191 ms per megapixel. Compared with conventional chemical staining workflows, the proposed virtual staining process can shorten the overall turnaround time by approximately 1–2 h. These results are summarized in Table S2. Also, the convergence curve of the proposed TAPO framework during training is given in Figure S14. The representative training curve shows a rapid loss decrease in the early stage, followed by a stable convergence trend, with the loss dropping from about 3.7 at the beginning to around 0.5 in the early training stage and gradually approaching about 0.1 in the later stage.

### 3.8 Loss Functions for Virtual Staining and Proxy Model Reconstruction

To optimize our virtual imaging framework, we employ customized loss functions tailored for two distinct processing pipelines:

(1) **Virtual Staining Loss**: We use Generative Adversarial Loss (GAN Loss) and Perceptual Loss to ensure realistic colorization and fine‐grained structural preservation. To enhance color realism and structural consistency, we employ a GAN‐based loss function where a generator **G** learns to transform an unstained image ***x*** = **I_ccd_** into a virtually stained image ***y*** = **G(I_ccd_)**, while a discriminator **D** distinguishes real stained images **I_gt_** from generated ones. With mathematical expectation **E**, the adversarial loss is defined as:

(4) $$L_{GAN}=E_{y}\left[\log D(y)\right]+E_{x}\left[\log(1-D(G(x)))\right]$$

To enforce high‐level structural similarity, we incorporate a VGG19‐based perceptual loss, which measures feature similarity at intermediate layers of a pre‐trained network:

(5) $$L_{percp}=\sum_{l}\lambda_{l}{\left\|\phi_{l}\left(G\left(x\right)\right)-\phi_{l}\left(y\right)\right\|}_{2}^{2}$$

where *ϕ_l_* denotes the activation of then *l*_th_ layer in VGG19. This loss preserves fine textures and tissue morphology beyond simple pixel‐wise similarity.

(2) **Proxy model Loss**: The output of our proposed virtual‐lens‐based proxy model needs to compute loss with real optical degradation measurements. Here, we adopt energy normalization loss 𝓛_EN_ and unsupervised saliency loss **𝓛**_saliency_ to emphasize global consistency. Since virtual lens imaging involves a mapping from a virtually stained domain to an imaging domain, we normalize the luminance consistency across the predicted and target images. We define the energy normalization loss as:

(6) $$L_{EN}={\left\|Y(G(x))-Y(y)\right\|}_{2}^{2}$$

where ***Y*(·)** extracts the luminance (Y) channel in the YUV color space. This stabilizes intensity variations, ensuring consistent brightness while allowing spectral variations. Additionally, we adopt a saliency‐based loss that aligns high‐level image structures to maintain structural consistency while allowing flexibility in low‐level details, thereby reducing learning complexity and focusing model capacity on another staining objective. Given a saliency map ***S*(*x*)**, the loss is defined as:

(7) $$L_{saliency}=\sum_{i,j}\left|S{\left(G(x)\right)}_{i,j}-S{\left(y\right)}_{i,j}\right|$$

The total loss function is formulated as:

(8) $$L=w_{GAN}L_{GAN}+w_{percep}L_{percep}+w_{EN}L_{EN}+w_{saliency}L_{saliency}$$

where different ***w*** values control the relative importance of each term, balancing realism, perceptual fidelity, and structural integrity.

## 4 Discussion

State‐of‐the‐art virtual staining systems often rely on fixed input contrasts or standard intensity‐only measurements, which may be sufficient for visual morphology but not always for robust tissue‐component discrimination, making the reconstruction more vulnerable to hallucination and reduced cross‐task adaptation. When staining goals change, these empirically fixed contrasts may fail to capture subtle yet critical cues. Here, we introduce a task‐adaptive plain‐to‐stain virtual staining system that jointly optimizes optical encoding and physics‐informed decoding within an end‐to‐end differentiable loop. At its core is the task‐adaptive optical acquisition with nontoxic illumination, enabling optics‐attention mechanisms to grade the optical contribution from each optical transfer stage, and making the segmentation clinically interpretable. This design enables independently learnable hardware encoding modes, allowing hierarchical hardware‐level attention that selectively enhances diagnostically relevant features rather than uniformly emphasizing all visible structures. By dynamically steering optical encoding based on downstream semantic objectives, TAPO achieves a hardware–algorithm synergy for high interpretability that is unattainable in prior static or post‐hoc correction frameworks. By embedding diagnostic objectives directly into the optimization, the system aligns physical acquisition with semantic interpretation, avoiding the mismatch between physically optimal and diagnostically optimal imaging conditions. The motivation of TAPO is therefore not only to improve the reconstruction network, but also to enrich the source measurements with task‐relevant spectral and phase‐related cues before virtual staining. The result is a unified plain‐to‐stain pipeline that actively adapts to task‐specific needs, reduces the degree of dependence on empirical parameter tuning, and delivers reproducible, interpretable, and clinically relevant virtual stains.

We extensively validated the proposed TAPO framework across multiple clinically relevant conditions, focusing on routine diagnostic imaging scenarios in terms of tissue type, staining protocol, imaging scale, and sample preparation, as well as introducing challenges such as tissue overlap caused by uneven dewaxing or mounting artifacts in thick sections, and reduced signal‐to‐noise ratio at high magnification. The convergence criterion and physics‐informed consistency constraints ensure that task adaptation is implemented as a physically constrained optimization process for obtaining stable task‐specific measurements. In all test conditions, the virtually stained outputs successfully preserved fine‐grained histological features. Remarkably, in thick (10 µm) and high‐magnification (20×) cases, the model maintained reconstruction quality under reduced illumination and defocus, benefiting from exposure‐adaptive acquisition and coherent field recovery. The present 10 µm study provides a useful basis for future extension toward substantially thicker specimens, including 50–100 µm and block‐level samples, where customized optical‐sectioning strategies such as light‐sheet‐based imaging will be further investigated. This enables comprehensive lesion profiling and spatially resolved biomarker localization in large biopsies. In comparison to unsupervised alternatives, our method consistently yields superior reconstruction fidelity and enhanced accuracy in nuclear localization, enabling more reliable diagnostic interpretation. Quantitative assessments demonstrated consistently low perceptual divergence (KID, FID) and high structural fidelity (PSNR, Dice scores), while statistical analyses of nuclei‐level features (count, spacing, diameter) confirmed accurate recovery of diagnostic markers. In addition, we further complemented the internal baseline experiments with a broader literature comparison against representative virtual staining methods based on unstained bright‐field microscopy, label‐free photoacoustic histology, autofluorescence microscopy, and FLIM, which further supports the structural fidelity and practical advantages of the proposed task‐adaptive optical encoding framework. TAPO accommodates a variety of diagnostic needs in clinics. Covering commonly used H&E and special Masson's trichrome, and it supports both general‐purpose morphological evaluation and collagen‐rich fibrosis‐specific visualization for connective tissue analysis.

TAPO's ability to generate rapid, high‐throughput, and highly consistent staining results will markedly shorten diagnostic turnaround times and enhance overall efficiency. Importantly, unlike traditional digital pathology workflows requiring manual conversion of glass slides into digital formats via scanners and often necessitate repeated sectioning and restaining for suboptimal samples, our system directly outputs interoperable quality virtual pathology images. Compared with conventional chemical staining, this design replaces the reagent‐based staining process with a task‐adaptive computational virtual staining process, while keeping the downstream staining quality evaluation and pathology diagnosis steps unchanged. This workflow can shorten processing time, avoid chemical labeling, and reduce phototoxicity, while still providing virtual stained images for routine pathological evaluation.

Despite these strengths, a practical limitation of TAPO lies in its training phase, which requires high‐refresh‐rate DMD hardware and stable optomechanical control to support efficient closed‐loop optimization. However, this requirement applies only during training: once the optical parameters have converged, the inference stage is entirely digital and produces virtual stains within milliseconds, making routine clinical deployment lightweight and hardware‐independent. Future work will address its extension to more complex and dynamic scenarios. These include real‐time virtual staining of living or freshly excised tissues, application to immunohistochemical (IHC) staining and organelle‐specific labeling, as well as inference of cellular states such as apoptosis, proliferation, or differentiation. Moving forward, integrating this hardware‐aware framework with super‐resolution imaging techniques may enable subcellular‐level virtual staining with enhanced morphological detail. Additionally, expanding into multimodal imaging (such as combining label‐free scattering, autofluorescence, or Raman contrast) offers the opportunity to jointly leverage complementary physical signals. This raises open questions on how to effectively fuse multi‐contrast information, design unified encoding strategies, and train cross‐modal networks that respect both spatial coherence and diagnostic interpretability. These directions will ultimately bridge virtual staining with functional and molecular pathology, enabling a new class of smart, standardized, and fully digital diagnostic platforms.

## Author Contributions

J.S. conceived the idea and supervised the research. D.J. supervised the research. T.H. developed the algorithm, captured and built the dataset, conducted the model training and testing, and wrote the manuscript. W.J. made tissue slides, performed the chemical staining, designed and conducted the experiment of blind evaluation with pathologists. L.Z. prepared the figures and wrote the manuscript. T.K. and W.J. conducted the experiment of quantitative characterization of tissue morphology. Y.W. and T.H. designed and fabricated the system, calibrated the sensors, and tested the imaging performance. Q.Z. and J.S. performed the theoretical analysis of optical staining and wrote the manuscript. H.Z. performed a stain efficacy assessment on optically stained images during the fine‐tuning of the algorithm. Z.Z. and J.S. performed the numerical simulations on optical imaging. All authors took part in designing the experiments.

## Funding

This work is founded by the National Key Research and Development Program of China (2022YFC2410102); Jiangxi Science and Technology Program (20224AAC01011); Sichuan Science and Technology Program (2025YFHZ0333).

## Conflicts of Interest

The authors declare no conflicts of interests.

## Supporting information




**Supporting File**: advs76292‐sup‐0001‐SuppMat.docx.

## Data Availability

The dataset specific to our optical staining of different organs is available upon request. The source code for model training and inference will be released promptly after publication.
