# Supplementary material for: Hardware‐Attentive Programmable Fourier Ptychography Enables Task‐Adaptive Label‐Free Virtual Staining
Source: Adv Sci (Weinh). 2026 Jun 30;13(46):e76292. doi: 10.1002/advs.76292 (PMC13336860; doi:10.1002/advs.76292)
Supplement: Supplementary file 1 [file ADVS-13-e76292-s001.docx]

Supplementary Materials for

**Hardware-Attentive Programmable Fourier Ptychography enables Task-Adaptive Label-Free Virtual Staining**

Tianyue He ^1^, Wenyi Jing^2^, Le Zhang^3^, Qican Zhang^1^, Tingdong Kou^1^, Xin He^2^, Yan Qiu^2^, Yang Lu^2^, Jian Cui^2^, Yongfu Wen^4^, Zhenrong Zheng^5^, Hongying Zhang^2^*, Dayong Jin^3^*, and Junfei Shen^1^*

^1^ College of Electronics and Information Engineering, Sichuan University, Chengdu, Sichuan, 610065, China

^2^ Department of Pathology, West China Hospital, Sichuan University, Chengdu, Sichuan, 610041, China

^3^ Institute for Biomedical Materials and Devices (IBMD), Faculty of Science, University of Technology Sydney, Ultimo 2007 New South Wales, Australia

^4^ Jiangxi Gaorui Optoelectronics Co., Ltd., Nanchang, Jiangxi, 330096, China

^5^ State Key Laboratory of Modern Optical Instrumentation, College of Optical Science and Engineering, Zhejiang University, Hangzhou, 310027, China

^*^Correspondence: [hy_zhang@scu.edu.cn](mailto:hy_zhang@scu.edu.cn), [dayong.jin@uts.edu.au](mailto:dayong.jin@uts.edu.au), [shenjunfei@scu.edu.cn](mailto:shenjunfei@scu.edu.cn)

**The PDF file includes:** Supplementary Text**,** Figs. S1 to S14, Table S1 to S2

Supplementary Text

**Strategy and Rationale behind Learnable Optical Parameter Design for Task-adaptive Encoding**

Unlike most prior differentiable imaging frameworks that focus on RGB-oriented image-quality optimization tasks such as chromatic aberration correction, field-of-view extension, extended depth of field, or super-resolution^1-3^, the present work aims at task-adaptive multimodal information extraction and reconstruction for label-free virtual staining. To enable efficient, adaptive acquisition of diagnostically relevant optical information, we design a learnable optical encoding scheme that jointly optimizes hardware-level parameters in coordination with downstream decoding objectives. Unlike conventional static imaging setups, our system leverages a task-aware end-to-end optimization pipeline, where each optical degree of freedom contributes to an integrated information encoding process. Central to this strategy is a physics-informed virtual lens (VL) proxy model, which provides a differentiable link between hardware configuration and image formation behavior. Within this framework, four categories of physical parameters are jointly optimized with network weights: illumination spectrum, angular direction, exposure time, and imaging distance. These parameters are not treated in isolation; instead, they form a cohesive encoding space in which spectral, spatial, and temporal domains are intelligently modulated according to task-specific demands.

**(1)** Spectral selection via DMD1 pattern optimization. The first digital micromirror device (DMD1) controls the illumination spectrum by selecting wavelength bands. Spectral modulation plays a foundational role in virtual staining, as different wavelengths interact with tissue components in distinct ways, reflecting intrinsic biochemical and structural variations^4^. Importantly, wavelength tuning enables implicit focal scanning due to chromatic dispersion, which supports extended-depth-of-field reconstructions. In our system, the learned spectral weights are not merely passive color filters but active encoders that adapt to the structural heterogeneity and optical absorption characteristics of each tissue sample. These wavelength-specific encodings synergize with angle and depth modulations to ensure that the optical system samples the most informative spectral content under constrained acquisition budgets.

**(2)** Angular spectrum modulation via DMD2 pattern optimization. The second DMD governs the illumination angle, directly influencing how spatial frequency content is projected into the imaging aperture. Prior studies have shown that adding optical information beyond standard bright-field intensity, such as phase and spectral cues, can necessarily improve AI-based tissue analysis. Our learnable angular encoding strategy shifts high-frequency components into the objective's passband and supports more accurate phase learning^5^. This modulation provides complementary structural information across captures and enriches the angular diversity of the coherent field. The network dynamically adjusts illumination angles to extract angular-frequency features, enabling robust subcellular feature recovery even in thick or optically complex samples. By leveraging task-driven angular diversity, the system encodes complementary optical responses across views, which enhances inverse reconstruction robustness under conditions of depth variation, tissue overlap, and scattering. When combined with spectral and temporal encoding, angular modulation enhances frequency coverage while reducing redundancy.

**(3)** Exposure time adjustment as a dynamic SNR regulator. Exposure time is modeled as a learnable scalar for each frame, enabling the system to adaptively regulate the signal-to-noise ratio (SNR) across diverse wavelength-angle illumination conditions. Unlike digital gain, which amplifies both signal and noise, modulating exposure time increases the collected photon count at the sensor, thereby enhancing signal strength from the physical acquisition level while maintaining noise stability. This adjustment compensates for the natural variability in incident light intensity and sample reflectance, equalizing the dynamic range across frames. As a result, the network avoids overexposure in strong-signal channels and preserves detail in low-signal ones, ensuring that high-frequency or subtle structural features are faithfully retained during training.

**(4)** Imaging distance as a depth-aware encoding factor. Tissue sections inherently possess structural variation along the axial direction, especially in thicker 10μm section preparations. A learnable imaging distance parameter allows the imaging system to focus at different sample depths, capturing volumetric cues otherwise missed by planar imaging. In this work, we selectively enabled optimization of the imaging distance (Δd) only for the 10μm thick sections, allowing the model to dynamically adapt its focal plane and better compensate for depth-induced aberrations. The learned Δd represents the shift distance from the ideal focal position, correcting for sample-induced focal drift during training. In contrast, Δd was fixed in the 4μm experiments, where tissue thickness posed minimal defocus risk. In addition, this parameter controls the system's effective PSF, influencing the coherence and blur characteristics of the captured field. By optimizing imaging distance within the training loop, the virtual lens proxy model learns to simulate depth-dependent wavefront propagation and capture the focused tissue information from different depths. This results in improved reconstruction fidelity across heterogeneous tissue layers, particularly in wide-field or high-magnification imaging tasks.

Beyond the individual roles of each parameter, the strength of our system lies in their joint modulation. Through coordinated spectral-angular encoding, temporal exposure time adjustment, and depth adaptation, the system dynamically shapes the incident light field to emphasize diagnostically relevant features while suppressing redundant or noisy components. This cooperative encoding strategy transforms each captured frame into a targeted information packet, optimized for both physical consistency and semantic saliency. During training, the system learns to generate time-variant structured illumination patterns that span the joint spectral-angular space. These encoded frames are then interpreted through our proxy-based forward model, which simulates optical propagation and guides the network’s inverse reconstruction. By constructing a coherent space-time-spectral representation of the tissue, our method balances the trade-off between spatial resolution, spectral bandwidth, and temporal efficiency. The result is an intelligent optical acquisition pipeline where each hardware degree of freedom serves as a task-adaptive encoder, jointly contributing to high-efficiency, label-free coherent information recovery. The combination of an optimizable optical-parameter matrix, hardware-attention mechanism, and physics-informed virtual-lens proxy model distinguishes the proposed framework from both RGB-oriented differentiable imaging and blind image-to-image virtual staining. The robustness of the framework is supported by validation across data collected from different experimental batches and tissue preparation times, different tissue types (liver and lung), pathological states (normal and lesion tissues), staining protocols (H&E and Masson’s trichrome), magnifications (10× and 20×), and section thicknesses (4μm and 10μm). Across these settings, the proposed method maintained stable virtual staining quality in tissue morphology, stain-like color appearance, and structural fidelity, and the generated results were further supported by pathologist evaluation, indicating that the learned encoding strategy is strongly robust to varied acquisition conditions and application scenes.

**Optical staining results discussion under 10× objective lens**

For model test, we began by evaluating the performance using unstained 4um liver tissue images obtained with 10×/0.1 NA objective lens (Fig. S1). Fig. S1(a) illustrates the transformation process from unstained microscopic images to virtual HE-stained images with several intermediate images. The restored H&E not only has an accurate aligned coloration with the reference, but also shows an improvement in high frequency components compared with the input images. Analysis of the detailed patches (rightmost subfigure) reveals that key pathological structures, including nuclear architecture, cellular morphology, and the surrounding extracellular matrix, are well-preserved. Hepatocytes are polygonal with centrally located, dark blue-stained nuclei, indicative of chromatin-rich regions. The cytoplasm displays a lighter pink hue, reflecting typical eosinophilic staining patterns. These hepatocytes are uniformly distributed, and their arrangement around central veins reflects preserved hepatic architecture. Sinusoidal spaces, appearing as pale areas among hepatocytes, represent vascular channels lined by endothelial cells and Kupffer cells. The high accuracy in replicating histological detail demonstrates the potential of virtual staining for diagnostic workflows. The reconstructed complex amplitude maps **I_amp_** highlight subcellular structural contrast, revealing nuclear-cytoplasmic boundaries and tissue-level textures with enhanced clarity. This improvement in nuclear visibility provides a physically meaningful prior that facilitates subsequent virtual staining, particularly in identifying fine-grained nuclear morphology essential for clinical interpretation. The input reconstruction **I_in_**, generated through the proxy-based forward simulation from the same wavefield, exhibits strong structural correspondence with the captured raw images **I_ccd_**, confirming physical consistency under the learned imaging configuration.

Notably, the virtually stained results **I_out_** (Fig. S1(a)) present sharper nuclear boundaries and finer texture than the chemically stained ground truth. This improvement comes from incorporating additional physical constraints during training. Unlike conventional approaches that rely solely on stained images as supervision, our framework also integrates raw unstained inputs and a physics-informed proxy model to provide additional training supervision. The stained images provide semantic guidance on tissue appearance and color distribution, while the raw unstained inputs act as a physical posterior constraint for high-fidelity coherent field reconstruction. This dual alignment guides the network to preserve meaningful fine structures that are often lost in traditional staining and produces virtually stained outputs that are both physically consistent and clinically interpretable.

Figure S1(c) illustrates the optimized proxy model optical parameters with virtual lens profile tailored for 4μm-thick liver tissue sections. The optimized wavelengths of DMD1 (546nm, 618nm, 570nm) enhanced spectral contrast for hemoglobin and other hepatic chromophores, offering greater sensitivity to subtle subcellular structures such as cytoplasmic granules. This optical optimization result suggests that our task-aware encoding strategy adaptively learns spectrally informative bands aligned with tissue-specific biochemical variation. The optimized DMD2 angular masks encode structural diversity through directional illumination, enabling multi-perspective contrast of hepatic plates and connective tissue boundaries. The exposure time and imaging distance are co-adapted to stabilize signal-to-noise ratio while maintaining axial sharpness across heterogeneous regions. The jointly optimized virtual lens surface exhibits a smoothly varying asymmetric phase profile, which can be decomposed into dominant Zernike components resembling defocus and astigmatism correction. The associated PSFs demonstrate concentrated central lobes with attenuated sidelobes, indicating enhanced focusing performance and defocus suppression, which provides a physical consistency with real imaging system for high-accuracy cellular detail reconstruction.

**Virtual Staining with Different Magnifications (20× objective lens)**

To assess the adaptability of our framework across resolutions, we conducted additional experiments at a higher magnification of 20× using liver tissue sections imaged with a 20×/0.2 NA objective. Compared to 10× magnification, the 20× setup provides approximately fourfold higher spatial sampling, enabling the capture of finer morphological features such as chromatin texture, nucleoli visibility, and cytoplasmic granularity. These structures are clinically significant for tasks such as nuclear grading and subcellular abnormality detection, which require more deliciated histological information. However, higher magnification introduces several new challenges. First, due to the reduced field of view, the total light collected per frame is significantly lower than that at 10×, resulting in decreased signal intensity. This reduced photon budget makes the images more vulnerable to noise, particularly in background and low-contrast regions. Second, the smaller field of view means that acquiring the same tissue area requires a greater number of frames, increasing both acquisition time and data volume. The effective field of view (FOV) per capture is significantly reduced, increasing the complexity of contextual understanding and potentially amplifying noise in low-contrast regions.

Therefore, at higher magnifications, the photon budget is severely constrained and more efficient encoding strategy is necessary. In this case, our method leverages hardware-aware adaptive encoding to extract diagnostically relevant information with improved efficiency. By jointly optimizing spectral, angular, and temporal acquisition parameters, the system reallocates limited optical resources toward signal-rich regions, ensuring high-fidelity reconstruction (Fig. S2(a)). In Fig. S2(b), metrics such as nuclear count accuracy, structural correlation, and perceptual similarity all show measurable gains at 20× compared to 10×. In Table 2, the KID and PSNR increase consistently with magnification, while FID remains stable, indicating that the gain in detail does not come at the cost of semantic distortion. This can be attributed to the task adaptive ability of our system maximizes the information transfer under fixed hardware constraints, enabling high-efficient utilization of high-frequency components at 20× magnification and achieving consistent reconstruction fidelity despite reduced field of view and increased noise susceptibility.

Fig. S2(c) shows the optimized optical parameters and virtual lens design for liver tissue under 20× magnification. Compared to 10×, the learned spectral bands (512nm, 643nm, and 696nm) coincide with spectral regions that show strong absorption contrast between liver parenchyma and vascular or fibrotic components. Specifically, these wavelengths enhance the differentiation of hemoglobin-rich sinusoidal regions and collagen deposition in fibrotic septa. This shift reflects the system’s task-adaptive behavior: at higher magnification, where the emphasis shifts to finer spatial details, the network prioritizes spectral encoding that enhances morphological discriminability at microstructural scales. The learned angular patterns diversify spatial sampling under the smaller FOV to prioritize high-frequency spatial content that matches the resolving power of the objective lens, helping to retain contextual continuity despite localized views. In particular, the model learns to extend exposure time selectively for critical views during training, compensating for reduced light throughput at 20× while maintaining acceptable acquisition efficiency.

**Optical staining results discussion for 10μm experiment**

Tissue thickness plays a critical role in optical imaging and directly affects the quality of virtual staining. Standard histological sections are typically 3~4μm thick to ensure adequate staining penetration and optical focus, but thin sections may fail to preserve full cellular morphology or the depth continuity of pathological features, especially in heterogeneous or lesion-rich tissue regions. Thicker sections, such as 10μm, offer a more complete representation of tissue architecture, including deeper nuclear features and multilayer cellular context, which are essential for accurate assessment in conditions like tumor boundary delineation and tissue infiltration analysis. However, the increased thickness leads to greater optical path length variation, pronounced defocus, stronger light scattering, and higher background signal from out-of-focus planes. These effects can degrade contrast, reduce spatial resolution, and make virtual staining more sensitive to errors in depth alignment.

To address these issues, we selectively enabled optimization of the imaging distance (Δd) only for the 10μm thick sections, allowing the model to dynamically adapt its focal plane and better compensate for depth-induced aberrations. The learned Δd represents the shift distance from the ideal focal position, correcting for sample-induced focal drift during training. In contrast, Δd was fixed in the 4μm experiments, where tissue thickness posed minimal defocus risk. Model performance on 10μm liver lesion tissue sections are given in Fig. S3(a). The 10μm lesion reconstructions reveal distinctive pathological features such as anisotropic spatial distribution and crowded chromatin architecture (hallmarks of malignancy that are difficult to capture in thin-section virtual staining). These results are particularly important for clinical scenarios requiring comprehensive depth information, such as detecting cellular pleomorphism, mitotic figures within layered structures, or tumor invasion at complex tissue boundaries. Fig. S3(b) shows the optimized optical parameters. For thick and structurally complex liver lesion sections, the model incorporates distance Δd (the relative distance compared to ideal focal location) as a learnable parameter to accommodate extended depth-of-field requirements. Nonzero optimized values of Δd (0.010, 0.006, –0.008mm) enable the proxy model to simulate defocus effects and reconstruct multiple in-focus layers. Exposure times across wavelengths (130, 75, and 15 ms) reflect adaptive signal accumulation under varying optical densities, enhancing SNR in deeper regions. The resulting PSFs exhibit broader spatial spread, favoring multi-layer integration and structural continuity, which is critical for recognizing malignancy-associated features embedded at different depths. Additionally, the learned optical parameters from 10μm tissue (PSF, spectral bands, and virtual lens configuration) exhibit notable differences from those optimized for 4μm tissue sections, indicating an adaptive adjustment of the imaging strategy based on tissue thickness.

Accurately distinguishing between normal and cancerous tissue is essential for histopathological diagnosis. Cancerous tissue often displays highly malignant nuclear morphology, with irregular shapes, enlarged sizes, and disorganized spatial arrangements. These structural abnormalities are typically accompanied by deeper staining intensity and greater intra-sample variation, which complicates the reconstruction process—particularly in thick sections where overlapping features and defocus further obscure fine details. The quantitative analysis in Fig. S3(c) provides further validation of our framework’s ability to distinguish cancerous and noncancerous regions at the nuclei level. In the 3D scatter plots, virtual staining (VHE) and ground truth (GT) both exhibit clear separability between lesion and normal tissues when projected in the feature space defined by nuclear count, mean diameter, and inter-nuclear spacing. The spatial distribution of points is consistent between VHE and GT, showing that the reconstructed images preserve diagnostically relevant nuclear features. The line plots (bottom) further confirm this trend across the image sequence. VHE closely follows GT in all three metrics: the number of nuclei is significantly higher in cancerous regions; the mean diameter shows greater variability in malignant areas, reflecting pleomorphism; and the inter-nuclear distance is markedly lower in cancerous tissue due to denser cellular arrangement. These accurate separations of nuclear-level features in thick tissue are enabled by our multi-dimensional adaptive optical encoding, which resolves defocus and depth misalignment through task-guided modulation of spectral, angular, and axial parameters, thereby preserving multilayer structures and enhancing the contrast and fidelity of deep pathological features critical for tumor boundary and infiltration analysis. In terms of color fidelity, the violin plots (top right) show that the pixel distributions in each RGB channel are well aligned between VHE and GT. The consistency in shape and median intensity suggests that the virtual staining output replicates the staining tone and intensity distribution of chemical H&E. The accompanying quantitative metrics support this: a low KID score (0.0025) and reasonable FID (48.63) demonstrate perceptual similarity, while the PSNR (18.57 dB) indicates adequate pixel-level fidelity.

Technically, our method successfully realizes normal/abnormal tissue classification and supports high quality thick tissue virtual staining. Accurate delineation of tumor infiltration depth, identification of invasive fronts, and preservation of spatial context are essential for staging malignant lesions, evaluating resection margins, and informing therapeutic decisions. By supporting reliable virtual staining and morphological assessment in thick sections, our approach helps bridge the gap between microscopic detail and large-scale tissue interpretation, advancing diagnostic precision in complex clinical scenarios such as large-volume biopsies and spatially resolved biomarker localization.

**Sample Preparation**

Formalin-fixed paraffin-embedded (FFPE) tissue sections with thicknesses of 4μm or 10μm were first deparaffinized and rehydrated, after which the unstained slides were imaged using the designed computational microscope. For hematoxylin and eosin (H&E) staining, the sections were immersed in hematoxylin solution for 5–7 minutes, differentiated briefly (1s) in acid-alcohol, rinsed in running tap water, and then transferred to distilled water. Bluing was performed in 49 °C warm water for 8 minutes, ensuring that nuclei appeared dark blue while the cytoplasm remained unstained. The slides were then stained with eosin for 8 minutes, followed by sequential rinses in tap water (3 minutes) and distilled water. Dehydration was carried out using 85%, 95%, and 100% ethanol, each for 3 seconds. After natural air-drying, the slides were mounted using a neutral, fast-drying mounting medium.

For Masson’s trichrome staining, the sections were first stained with iron hematoxylin for 5-10 minutes, followed by reinforced green dye for 1-2 minutes. A brief wash with a weak acid solution was performed for 30 seconds. Rapid dehydration was conducted in 95% ethanol for 2-3 seconds, followed by two rounds of dehydration in absolute ethanol (5-10 seconds each). The slides were then cleared twice in xylene (1-2 minutes each) and mounted using neutral balsam.

The dataset acquisition workflow starts from standard FFPE section preparation, followed by imaging of the unstained slides with the proposed system and subsequent chemical staining to generate paired reference images. This study was approved by the Ethics Committee on Biomedical Research, West China Hospital of Sichuan University (2025-1564). All samples were archival materials collected prior to this study, without any influence on clinical procedures or patient care. All identifiable patient information was removed before analysis, and no additional tissue collection was performed specifically for this research.

**Virtual Lens PSF formation model**

We provide a detailed derivation of Eq. (2) in the main text. It describes how to model the entire PSF formation process based on diffraction theory. Consider a point source produces a spherical wave that arrives at the incident plane of the virtual lens. The phase of complex-valued wave field is delayed by the optical element. Suppose that *U*_0_(*x_p_*, *y_p_*, *z*, *λ*) and *φ*(*x_p_*, *y_p_*, *λ*) separately represent the incident complex-wave field and delayed phase map of the virtual lens. The wave field right after it passed through the optical element is given by:

 (S1)

where *U*_0_(*x_p_*, *y_p_*, *z*, *λ*) = exp [*ik* (*x_p_*^2^+*y_p_*^2^+*z*^2^)^1/2^], *k* = 2π/*λ* is the wave number and *λ* is wavelength. (*x_p_*, *y_p_*) is the spatial coordinate at virtual lens plane, *z* is the object distance, and *A*(*x_p_*, *y_p_*) is a **circ** function with discretized value of zero and one that represents the aperture of the virtual lens. Before reaching a sensor, the output field *U_p_*(*x_p_*, *y_p_*, *λ*) then propagates in free space by a distance *d*. According to the scalar diffraction theory, the measured complex-wave distribution at sensor plane can be calculated by:

$U\left(x,y,\lambda\right)=\frac{e^{ikd}}{i\lambda d}\iint U_{p}\left(x_{p},y_{p},\lambda\right)e^{\frac{ik}{2d}\left[\left(x-x_{p}\right)^{2}+\left(y-y_{p}\right)^{2}\right]}dx_{p}dy_{p},$ (S2)

where (*x*, *y*) is the spatial coordinate at real sensor plane. As such, the PSF of the virtual lens *PSF_p_*(*x*, *y*, *λ*) is expressed as:

$PSF_{p}\left(x,y,\lambda\right)\propto\left|U\left(x,y,\lambda\right)\right|^{2}=\left|\frac{e^{ikd}}{i\lambda d}\iint U_{p}\left(x_{p},y_{p},\lambda\right)e^{\frac{ik}{2d}\left[\left(x-x_{p}\right)^{2}+\left(y-y_{p}\right)^{2}\right]}dx_{p}dy_{p}\right|^{2}.$ (S3)

**Multi-spectral and Super-resolution Imaging Ability**

Spectral imaging presents a fundamental challenge in computational pathology, where the goal is to recover spatially resolved intensity distributions across a broad wavelength range. This task demands a careful balance between photon efficiency, acquisition speed, and spectral resolution. Traditional hyperspectral microscopy either performs sequential narrowband scanning, which incurs significant time cost and phototoxicity risk, or relies on broadband illumination that leads to spectral crosstalk, low signal-to-noise ratios (especially in the UV and red edges), and reduced channel separability. These trade-offs make spectral reconstruction an ideal benchmark to assess the system’s task-aware adaptability and information-aware encoding capacity under varying physical constraints and output dimensionality. This challenge makes spectral reconstruction an ideal benchmark for evaluating our system's information-optimized encoding capabilities.

In this experiment, the system reconstructed a full spectral cube of 31 discrete bands ranging from 400 nm to 700 nm at 10 nm intervals, offering fine spectral granularity suitable for high-throughput spectral analysis. For clarity, only 8 representative bands are visualized in Fig. S7, but the underlying reconstruction covers the entire visible spectrum. To handle such a demanding task, the proposed system leverages hardware-level attention to enrich spatial frequency content across wavelengths. Each wavelength-angle combination acts as a distinct modulation pattern in both spectral and spatial domains, effectively encoding latent high-frequency details. Moreover, exposure time is treated as a learnable parameter for each spectral channel, enabling signal-specific SNR balancing. Unlike uniform digital gain, which amplifies both signal and noise, exposure-based adjustment introduces physically meaningful integration time, enhancing weak but diagnostically relevant signals without sacrificing fidelity. This is especially important given the natural variation in spectral reflectance across tissue types: certain bands such as 400-420 nm, and 680-700 nm, where illumination is weaker or detector sensitivity drops, pose significant challenges for accurate reconstruction. Nevertheless, our system demonstrates precise recovery even in these low-SNR regions in Fig. S7(a), as seen from the tight histogram overlap and low residuals across the full dynamic range. The reconstructed intensities closely match the ground truth distributions from short to long wavelengths, confirming that both spectral contrast and absolute intensity scales are faithfully preserved.

In terms of spatial detail, the recovered spectral images reveal fine textures and edge sharpness beyond what broadband imaging or naïve interpolation can provide. The system effectively resolves high-frequency variations across wavelengths, reflecting its capacity for spectral super-resolution with spatial enhancement. This ability to capture rich spectral-spatial correlations with minimal input channels establishes a scalable foundation for future high-dimensional imaging tasks, such as multiplexed biomarker localization or reflectance-based metabolic imaging.

The error maps in Fig. S7(b) further validate spatial reconstruction accuracy. Most areas exhibit sub-0.1 residuals, indicating agreement with ground truth. Residuals are generally uniform, but minor localized deviations appear in textured regions with fine gradient variations, such as around bright puncta or edge-rich zones (Fig. S7(c)). These may reflect the inherent difficulty of preserving both low-frequency contrast and high-frequency detail simultaneously in under-sampled spectral recovery. Notably, the absence of band-specific bias or structured artifacts confirms that our method avoids mode collapse or spectral drift, which could be seen as common issues in low-shot reconstruction.

Technically, this spectral reconstruction task highlights several key strengths of our framework. First, the ability to support multi-frame, overlapping spectral encodings under constrained measurement budgets demonstrates the scalability of the information-aware encoding mechanism. Second, the adaptive exposure strategy contributes to wavelength-wise signal balancing, ensuring that weaker bands are not overwhelmed in the fusion process. Third, the embedded optical proxy model enables physically consistent disentanglement of overlapping spectral responses, maintaining coherence across the spectral dimension. These features collectively form a robust solution to spectral super-resolution, pushing the boundaries of compact, data-efficient, and task-adaptive optical imaging systems.

**Network Architecture**

As outlined in the main text, our method adopts an adversarial generative framework tailored for coherent-field reconstruction. In the following, we provide a detailed description of the internal structure and design rationale of both the optical information recovery network and the discriminator components.

**Optical information recovery network.** The optical information recovery network, as depicted in Fig. S8, is designed as a generator to reconstruct spectrally encoded, phase-resolved complex wavefields information from multiple captured grayscale inputs. Structurally, it adopts an encoder-decoder framework with symmetric skip connections to preserve spatial details and enhance feature reuse. The encoder consists of stacked 3×3 convolutional layers, each followed by batch normalization and ReLU activation, interleaved with downsampling operations to progressively extract hierarchical representations. The decoder mirrors this structure with upsampling layers, which are followed by convolution, normalization, and activation blocks to refine the decoded features. The network outputs a 2N-channel tensor, which is further decomposed into N amplitude and N phase components, representing the complex-valued spectral wavefield across N spectral bands. All activation functions and intermediate operations are explicitly given in the architectural diagram to improve network interpretability. This complex-valued representation serves as the input for downstream virtual staining and enhances the network's capacity to model coherent imaging characteristics, including phase retrieval and spectral super-resolution.

**Discriminator**. The discriminator follows a VGG architecture to enforce high-frequency realism and texture consistency in the generated stained images. It comprises a series of convolutional layers with increasing channel dimensions (64 → 128 → 256 → 512), interleaved with LeakyReLU activations. Each convolution operates with stride 2 to downsample spatial dimensions while preserving discriminative features. The final convolution reduces the features to a single-channel probability map, providing patch-wise authenticity scores.

**Clinical Evaluation Criterion Design**

Different criterions are designed for pathologist blind evaluation:

**Clarity and Resolution** reflect the ability to resolve subcellular details such as nuclei, chromatin, and tissue boundaries, which are essential for accurate diagnosis.

**Image Noise and Distortion** impact visual comfort and structural accuracy. High noise may obscure fine features, while distortion can affect morphological interpretation.

**Staining Uniformity** ensures that tissue components are consistently visualized across the field of view.

**Hematoxylin and Eosin Intensities** determine nuclear-cytoplasmic contrast and are vital for distinguishing cell types and pathology-specific features.

**H&E Contrast Ratio** quantifies the perceptual differentiation between hematoxylin- and eosin-stained regions.

**Tissue Arrangement** reflects the integrity of overall tissue architecture and spatial coherence between structures.

**Cellular Morphology** is a holistic indicator combining shape, boundaries, and contextual positioning.

**Cytoplasmic Staining**, which supports the identification of cell type and functional state.

**Nuclear Staining** reflects the clarity and contrast of nuclear regions relative to surrounding cytoplasm, which is crucial for assessing cellular identity and differentiating malignancies.

**Nuclear Resolution** evaluates the level of detail in nuclear morphology, including boundary sharpness and chromatin texture.

**Nuclear-Cytoplasmic Contrast (NCC) and Nucleolus Clarity** are considered fine-detail metrics that impact the ability to assess subnuclear features and cell state.

**Comparisons to different virtual staining baselines**

In Table S1, we further added a broader literature comparison with representative published virtual staining methods based on unstained bright-field microscopy, label-free photoacoustic histology, autofluorescence microscopy, FLIM, and other enriched-input settings. Overall, the added table shows that our method achieves the lowest reported FID and best SSIM value in this comparison. Since lower FID indicates better agreement between generated and reference image distributions, and higher SSIM indicates better structural similarity, these results suggest that our proposed TAPO achieves a strong balance between distribution-level realism and structural fidelity. Compared with the bright-field image-to-image methods, the advantage of TAPO is mainly related to the information contained in the input measurements. Methods such as Khan et al. ^6^ and Zhang et al. ^7,8^ use fixed unstained bright-field input and mainly rely on supervised, data-driven image translation without explicit optical priors. Their results show that standard bright-field images can support basic virtual staining, but the reconstruction is still limited by intensity-only input and data-driven learning. In contrast, TAPO introduces spectral and phase-related information through programmable visible-light optical encoding and constrains the reconstruction with a physics-informed proxy model. Results show that these additional cues are more directly useful for distinguishing stain-relevant tissue structures. This helps explain why TAPO performs better than the bright-field methods summarized in the table.

Methods such as Yoon et al.^9^, Yang et al. ^10^, and Wang et al. ^11^ already use richer input contrast than standard bright-field imaging, including photoacoustic contrast, fluorescence, or lifetime imaging. However, these methods introduce different practical trade-offs, such as UV excitation or fluorescence-related phototoxicity. In comparison, TAPO achieves the best SSIM and FID in the summarized comparison while operating under programmable visible-light encoding without phototoxic excitation. This indicates that TAPO can obtain high-quality virtual staining results without relying on high-energy or fluorescence-based excitation, due to the delicate optical codec design.

We further compared the phototoxicity, the input setting, and whether the framework is task-adaptive. Overall, the improvement of TAPO comes from both richer information acquisition and task-adaptive use of this information. By introducing spectral and phase-related cues, the input measurements contain more information useful for distinguishing subtle tissue structures. By further combining programmable optics with task-adaptive optimization, the system avoids exhaustive high-dimensional acquisition and selectively captures the most useful measurements for the staining task. Therefore, TAPO improves both the amount and the efficiency of task-relevant information acquisition, which supports better virtual staining fidelity and lower distributional divergence.

**Fig. S1. Virtual staining intermediate results and quantitative analysis on 4μm liver tissue under 10× magnification.** **(a)** Virtual H&E staining results, subcellular structural recovery, and proxy-model consistency on liver tissue. **Top-left:** representative unstained images (I_ccd_) captured under learned spectral-angular illumination. **Top-middle:** input reconstruction (I_in_) from the proxy-model degradation branch, demonstrating physical consistency. **Top-right:** recovered amplitude component (I_amp_) extracted from the coherent field W_spe_, revealing subcellular structural contrast. **Bottom-left:** virtually stained output (I_out_) from our model alongside the ground-truth H&E-stained image (I_gt_). **Bottom-middle:** H&E stained Ground-truth image. **Bottom-right:** high-resolution crops from three distinct tissue regions (nucleus-rich, texture-rich, and boundary) across all outputs. **(b)** Quantitative validation of virtual staining fidelity using ground truth references. (i) 3D scatter plot comparing nuclear statistics—including count, mean diameter, and mean spacing—between ground truth (GT) and virtually stained TAPO results. (ii) Per-frame trend plots of nuclear metrics across the image sequence further demonstrate structural consistency over space. (iii) Violin plots of RGB pixel value distributions for VHE and GT images reveal similar color histograms across all channels. (Table 1) quantitative metrics confirm high perceptual similarity (KID = 0.0071), competitive realism (FID = 48.08), and acceptable signal fidelity (PSNR = 17.73 dB). **(c)** Optimized spectral-angular illumination patterns and virtual lens design learned via end-to-end training. Left: three DMD1–DMD2 pattern pairs, corresponding to optimized spectral wavelengths (546 nm, 618 nm, 570 nm) and angular illumination masks. Each pair is associated with an optimized exposure time *t*. Right: reconstructed surface shape of the wavelength-specific virtual lens, parameterized using Zernike polynomials and visualized as a height map. Corresponding intensity PSF represent the system optical response, with PSF_r_ and PSF_i_ representing the real/imaginary part of the coherent PSF.

**Fig. S2.** **Virtual staining performance on 4μm liver tissue under 20× magnification.** **(a)** Visualization of the virtual staining pipeline applied to 20× liver tissue. **(b)** Quantitative comparison with histological ground truth across structural and spectral metrics. **(c)** Optimized hardware parameters and virtual lens configuration for the 20× setup.

**Fig. S3. Virtual staining and diagnostic evaluation on 10μm liver lesion tissue sections. (a)** Virtual staining and coherent Field recovery at 10μm thickness and lesion tissues. **(b)** Optimized hardware parameters and virtual lens configuration for the 10μm liver lesion tissue. To handle thick, complex liver lesions, the model learns a relative defocus distance (Δd), enabling multi-layer reconstruction. Optimized Δd values (0.010, 0.006, –0.008 mm) simulate defocus, while wavelength-specific exposures (130, 75, 15 ms) enhance SNR at depth. The broadened PSFs support structural continuity across layers, aiding malignancy detection. **(c)** Quantitative validation of diagnostic morphology and color fidelity. (i) The 3D scatter plots show clear clustering between cancerous (red) and noncancerous (blue) regions based on nuclear count, mean diameter, and spacing. (ii) Color distribution comparison confirms consistent intensity profiles across RGB channels, and image quality metrics (KID: 0.0025, FID: 48.63, PSNR: 18.57) indicate high structural fidelity and low perceptual divergence. (iii) The line plots trace three types of nuclear statistics across spatial sequences, further confirming the model’s ability to preserve diagnostic variation across heterogeneous tissue regions. These results demonstrate the method’s effectiveness for high-fidelity staining and morphological analysis in clinically challenging thick-tissue scenarios.

Fig. S4. Schematic of the multi-wavelength and multi-angle imaging system. (a) The custom-designed imaging setup integrates spectral and angular modulation through two digital micromirror devices (DMDs). DMD1 enables programmable wavelength selection across the visible spectrum, while DMD2 controls multi-angle illumination over 360°. A tunable 4f optical system with cone lenses generates annular illumination patterns of adjustable radius. The system supports variable magnifications and three-dimensional scanning, enabling flexible data acquisition for end-to-end learnable optical staining. Downstream of the illumination module, the transmitted light passes through a rotating objective turret for magnification switching, a tube lens for image formation, and a motorized XYZ translation stage for volumetric scanning. These modules form a coherent and reconfigurable optical acquisition pipeline, enabling high-dimensional light-field capture tailored to the virtual staining task. (b) Visualization of DMD modulation. The colored bars and circles represent different modulated light patterns projected onto DMD1 and DMD2, respectively. Both DMDs have a resolution of 1920 × 1080. White pixels (value = 0) indicate “not selected,” while gray pixels (value = 1) indicate “selected.” Only the selected pixels permit the corresponding spatial components of light to remain in the illumination path. In the example demonstration shown in (b), DMD1 selects the red and purple spectral bands, while DMD2 selects four distinct illumination angles, which enables fast and accurate modulation of illumination wavelength and angle.

**Fig. S5.** End-to-End optimization algorithm to realize hardware-attention training. The proposed framework jointly optimizes the imaging hardware parameters (illumination wavelengths ***λ***, illumination angles ***ω***, exposure time ***t***, and imaging distance ***d***) and the network parameters ***θ*** through backpropagation. During training, pyramid fusion is used to construct large depth-of-field stained references from multi-focus whole-slide images (details see Fig. S6). Based on the current parameter values, structured illumination patterns are dynamically generated by DMDs, and corresponding unstained images are captured. These images are aligned with ground truth and passed through the model to jointly optimize virtual staining outputs and proxy-model-based input reconstructions. Hardware attention is achieved by embedding physical acquisition parameters as learnable variables in the training loop, enabling the optical system to adaptively prioritize diagnostically useful features. This unified soft–hardware optimization pipeline ensures that both image acquisition and interpretation evolve together toward the target staining task.

Fig. S6. Generation of a large depth-of-field ground-truth image using pyramid fusion across five focal layers. The virtually stained reconstruction aligns well with the fused reference in both spatial structure and color appearance, validating the method’s robustness to axial defocus and thickness-induced blur.

**Fig. S7. Multispectral and super-resolution reconstruction and error analysis.** **(a)** Visualization of the recovered multispectral complex amplitude images across different wavelengths, compared against the corresponding ground truth spectral slices. **(b)** Error distribution histograms for selected wavelengths, where the x-axis represents the absolute error magnitude and the y-axis denotes probability density. **(c)** Spatial error maps corresponding to the reconstructed spectral slices, showing the absolute error between reconstructed and ground-truth amplitudes at each pixel.

**Fig. S8.** Optical information recovery network architecture demonstration.

**Fig. S9.** Integration of the proposed task-adaptive staining framework into the clinical pathology workflow.

**Fig. S10.** Preservation of nuclear integrity in thicker sections compared with thin-section truncation, where detected cell nuclei are delineated with blue/orange contours on the plain transmittance image. A substantial degree of nuclear fragmentation is observed in the 4µm sections, which may result in missed detections during segmentation. In contrast, most nuclei in the 10µm sections remain structurally intact.

**Fig. S11.** Workflow for paired-data generation and full-field scanning.

**Fig. S12.** Chemical staining workflow used for obtaining the stained ground-truth data.

**Fig. S13**. Training and convergence workflow for task-specific optical-parameter optimization.

**Fig. S14.** Convergence curve of the proposed TAPO framework during training.

**Table S1.** **Comparisons to different virtual staining baselines. (The best results are highlighted in bold red)**

| **Study** | **Input** | **Output** | **Input size** | **SSIM** | **FID** | **Task-adaptive** | **Phototoxicity** |
| --- | --- | --- | --- | --- | --- | --- | --- |
| Khan et al.^6^ | Unstained bright-field microscopy | H&E | 512×512 | Pix2pix 0.72  Dense-conv 0.75 | Not reported | No | **No** |
| Yoon et al.^9^ | Label-free photoacoustic histology (PAH, UV-PAM) | H&E | 512×512 | Not reported | CycleGAN 67.76  E-CycleGAN 61.91  CUT 54.87  E-CUT 50.91 | No | Yes, UV laser excitation |
| Zhang et al.^7^ | Unstained bright-field microscopy | H&E  PSR  EVG | 512×512 | H&E 0.58  PSR 0.71  EVG 0.58 | Not reported | No | **No** |
| Zhang et al.^8^ | Unstained bright-field microscopy | H&E | 512×512 | 0.53 | Not reported | No | **No** |
| Yang et al.^10^ | Multi-channel autofluorescence microscopy | Congo red  Birefringence | 2048×2048 | 0.7131 **±** 0.0837 | 86.7925 | Partial modality selection only | Yes, fluorescence excitation |
| Wang et al.^11^ | FLIM: autofluorescence lifetime + intensity | H&E | 256×256 | Intensity 0.689  α-FLIM 0.695  IW-FLIM 0.697 | Not reported | No | Yes, fluorescence excitation |
| **Ours (TAPO)** | Task-adaptive programmable visible-light optical encoding | H&E  Masson | 960×960 | **0.7561** | **liver H&E 48.08**  **liver lesion H&E 48.63** | **Yes** | **No** |

**Table S2. Summary of dataset composition and** **image acquisition settings.**

| Number of patients | 105 patients |
| --- | --- |
| Number of slides | 115 corresponding slides |
| Number of image groups | Over 35,000 groups of images |
| Tasks | Normal liver, lesion liver, lung, and Masson-stained liver |
| Raw image size | 4800 × 2880 pixels |
| Network input size | Cropped into 960 × 960 patches |
| Camera pixel size | 2.4μm |
| Field of view at 10× | 1.15 × 0.69 mm |
| Field of view at 20× | 0.58 × 0.35 mm |
| Train/validation/test split | 8:1:1 |
| Acquisition time for 10×/20×liver tissue | ~33/43 ms per frame |
| Training time | ~9 h per task |
| Inference time | ~191 ms per megapixel |

**Reference:**

1	Sitzmann, V. *et al.* End-to-end optimization of optics and image processing for achromatic extended depth of field and super-resolution imaging. *ACM TOG* **37**, 1-13, doi:10.1145/3197517.3201333 (2018).

2	Tseng, E. *et al.* Differentiable Compound Optics and Processing Pipeline Optimization for End-to-end Camera Design. *ACM transactions on graphics* **40**, 1-19, doi:10.1145/3446791 (2021).

3	Wang, C., Chen, N. & Heidrich, W. dO: A Differentiable Engine for Deep Lens Design of Computational Imaging Systems. *TCI* **8**, 905-916, doi:10.1109/TCI.2022.3212837 (2022).

4	Ortega, S., Halicek, M., Fabelo, H., Callico, G. M. & Fei, B. Hyperspectral and multispectral imaging in digital and computational pathology: a systematic review [Invited]. *Biomed Opt Express* **11**, 3195-3233, doi:10.1364/BOE.386338 (2020).

5	Kandel, M. E. *et al.* Phase imaging with computational specificity (PICS) for measuring dry mass changes in sub-cellular compartments. *Nat Commun* **11**, 6256-6210, doi:10.1038/s41467-020-20062-x (2020).

6	Khan, U., Koivukoski, S., Valkonen, M., Latonen, L. & Ruusuvuori, P. The effect of neural network architecture on virtual H&E staining: Systematic assessment of histological feasibility. *Patterns (N Y)* **4**, 100725, doi:10.1016/j.patter.2023.100725 (2023).

7	Zhang, G. *et al.* Image-to-Images Translation for Multiple Virtual Histological Staining of Unlabeled Human Carotid Atherosclerotic Tissue. *Mol Imaging Biol* **24**, 31-41, doi:10.1007/s11307-021-01641-w (2022).

8	Zhang, G. et al. Self-Attention Based Virtual Staining for Bright-field Images of Label-free Human Carotid Atherosclerotic Plaque Tissue Section. Annu Int Conf IEEE Eng Med Biol Soc, 3492-3495, doi:10.1109/EMBC46164.2021.9630026 (2021).

9	Yoon, C. *et al.* Deep learning-based virtual staining, segmentation, and classification in label-free photoacoustic histology of human specimens. *Light: Science & Applications* **13**, 226, doi:10.1038/s41377-024-01554-7 (2024).

10	Yang, X. *et al.* Virtual birefringence imaging and histological staining of amyloid deposits in label-free tissue using autofluorescence microscopy and deep learning. *Nat Commun* **15**, 7978-7917, doi:10.1038/s41467-024-52263-z (2024).

11	Wang, Q. *et al.* Deep learning-based virtual H& E staining from label-free autofluorescence lifetime images. *npj Imaging* **2**, 17-11, doi:10.1038/s44303-024-00021-7 (2024).
